# Interdisciplinary fetal-neonatal neurology training improves brain health across the lifespan

**DOI:** 10.3389/fneur.2024.1411987

**Published:** 2024-07-04

**Authors:** Mark S. Scher

**Affiliations:** Department of Pediatrics and Neurology, Division of Pediatric Neurology, Fetal/Neonatal Neurology Program, Case Western Reserve University School of Medicine, Cleveland, OH, United States

**Keywords:** interdisciplinary fetal-neonatal neurology training, neural exposome, developmental origins, life course, brain health, healthcare disparities, diversity-equity-inclusion, global educational synergy

## Abstract

Integrated fetal, neonatal, and pediatric training constitute an interdisciplinary fetal-neonatal neurology (FNN) program. A dynamic neural exposome concept strengthens curriculum content. Trainees participate in mentoring committee selection for guidance during a proposed two-year program. Prenatal to postnatal clinical learning re-enforces early toxic stressor interplay that influences gene–environment interactions. Maternal-placental-fetal triad, neonatal, or childhood diseases require diagnostic and therapeutic decisions during the first 1,000 days when 80 % of neural connections contribute to life-course phenotypic expression. Pediatric follow-up through 3 years adjusts to gestational ages of preterm survivors. Cumulative reproductive, pregnancy, pediatric and adult exposome effects require educational experiences that emphasize a principle-to-practice approach to a brain capital strategy across the lifespan. More rigorous training during fetal, neonatal, and pediatric rotations will be offered to full time trainees. Adult neurology residents, medical students, and trainees from diverse disciplines will learn essential topics during time-limited rotations. Curriculum content will require periodic re-assessments using educational science standards that maintain competence while promoting creative and collaborative problem-solving. Continued career-long learning by FNN graduates will strengthen shared healthcare decisions by all stakeholders. Recognition of adaptive or maladaptive neuroplasticity mechanisms requires analytic skills that identify phenotypes associated with disease pathways. Developmental origins and life-course concepts emphasize brain health across the developmental-aging continuum, applicable to interdisciplinary research collaborations. Social determinants of health recognize diversity, equity, and inclusion priorities with each neurological intervention, particularly for those challenged with disparities. Diagnostic and therapeutic strategies must address resource challenges particularly throughout the Global South to effectively lower the worldwide burden of neurologic disease. Sustainable development goals proposed by the World Health Organization offer universally applicable guidelines in response to ongoing global and regional polycrises. Gender, race, ethnicity, and socio-economic equality promote effective preventive, rescue and reparative neuroprotective interventions. Global synergistic efforts can be enhanced by establishing leadership within academic teaching hubs in FNN training to assist with structure and guidance for smaller healthcare facilities in each community that will improve practice, education and research objectives. Reduced mortality with an improved quality of life must prioritize maternal-pediatric health and well-being to sustain brain health across each lifespan with transgenerational benefits.

## The neural exposome redefines neuroplasticity

Professional societies and health policy organizations now strongly advocate for life-course brain health (1, 2). This objective can be more effectively achieved by recognizing the continuity of reproductive, pregnancy and pediatric exposome effects that influence a dynamic neural exposome across each person’s lifespan (3). The epidemiological concept of the exposome was first introduced to diagnose and treat cancer based on the totality of lifetime exposures with gene–environment (G × E) interactions (4). This approach is now applicable to improve neurodiagnostic, neurotherapeutic and prognostic decisions by considering a life-course neural exposome perspective regarding brain health (5). Formal training is followed by career-long re-application of neurological principles and practice to facilitate the maintenance of brain health for all persons across the lifespan.

An interdisciplinary fetal-neonatal neurology (FNN) educational approach is proposed that will help advance precision-medicine to improve life-course brain health. Supervised clinical encounters by trainees offer strategies to avoid or minimize disease effects that represent early life maladaptive neuroplasticity. Two surveys regarding training experiences in fetal-neonatal medicine stress the need for this specific subspecialty training. An earlier report was based on multiple pediatric subspecialist responses followed by a more recent survey specifically directed to pediatric neurologists. The earlier study results concluded that pediatric neurologists received the least training in FNN. The more recent survey completed by pediatric neurologists indicated their preference to acquire enhanced educational experiences in this subspecialty (6, 7).

Ontogenetic adaptation (OA) is a robust evolutionary principle (8) applicable to all biological disciplines across the developmental-aging continuum. Relevance of past experiences improve or impair future structure and function through multisystemic effects. This concept can assist all neurology subspecialists to better recognize phenotypic expressions that reflect positive or negative neuroplasticity across each lifespan. Clinical signs that represent dynamic OA changes occur during critical/sensitive time periods starting with the first 1,000 days. This interval encompasses reproductive, pregnancy and early life experiences until 2 years of age during which 80% of neuronal connections have been established (9, 10). Multisystemic adaptive responses to disease and adversity promote survival and positively influence brain connectivity across advancing developmental-aging stages. Earlier adaptive changes respond to later adverse experiences by minimizing or avoiding negative consequences. Improved outcomes result in response to positive stressor effects. Maladaptation alternatively may occur based on the intensity and specific combination of stressor effects that limit or reverse earlier positive modifications. Multiple negative encounters promote permanent maladaptive neuroplasticity which will later be expressed across the lifespan as neurological dysfunctional phenotypes (11), sometimes identified with structural brain lesions. These abnormal phenotypes may be expressed initially as fetal, neonatal, or early childhood neurologic disorders, or alternatively appear more remotely during the adult stage of the life cycle.

**Figure 1:**
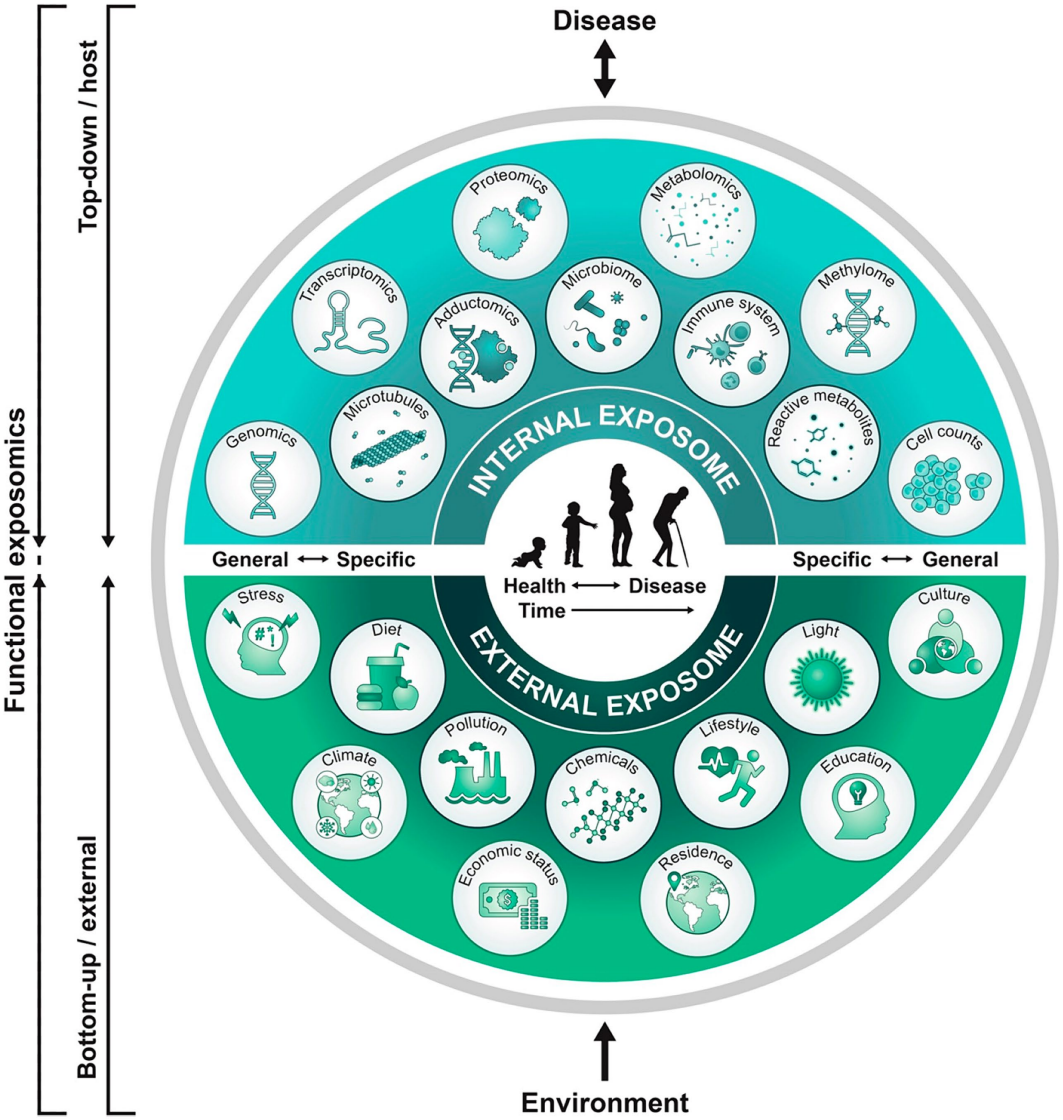
A functional exposome approach across the lifespan is illustrated that is applicable to the neural exposome (11). A top-down approach uses molecular epidemiological studies focused on biologic response profiles associated with identified xenobiotic exposures using “omics” technologies that analyze host biospecimens. This approach generates hypotheses regarding exposure-disease and exposure-response relationships without necessarily capturing direct measures of exposure. Applying the bottom-up approach, comprehensive data on environmental exposures are collected through surveys, sensors, or trace chemical analyses of biospecimens to generate hypotheses on effects without necessarily investigating a specific effect on the host. This functional exposome perspective bridges both approaches to improve an understanding of the expression of brain health or disease. A functional neural exposome is similarly comprised of biologically active exposures that influence the nervous system through adaptive or maladaptive neuroplasticity mechanisms. Serial evaluations assess associations among environmental exposures and biological effects on the brain health or disease across development and aging.

Endogenous and exogenous toxic stressor interplay (TSI) exert life-long harmful effects through G × E interactions in response to communicable and non-communicable illness and adversity (3). This functional exposome concept is applicable to a dynamic neural exposome that will change expressions across time (Figure 1) (11). Abnormal form and function result from cumulative TSI effects that represent evolving maladaptive neuroplasticity responses over the lifespan. Adverse effects are first expressed by women’ s health status before each pregnancy. These abnormal diseases or adversities subsequently impair maternal-placental-fetal (MPF) triad health. Parental and familial inherited conditions contribute to fetal genetic expressions are further altered by post-translational acquired disease processes representing dysregulatory neuronal changes at any time across pregnancy. Childhood followed by adulthood neurologic phenotypes are later expressed, unique to each person’s dynamic neural exposome. Transgenerational effects perpetuate (12) neurological disease presentations. Diagnostic skills are needed that preserve brain health by reducing negative effects from multiple stressors before and during each person’s lifespan by selecting the most effective neuroprotective interventions. Prolonged survival with an improved quality of life are therefore dual objectives required for the education of all neurology subspecialists.

Equitable healthcare delivery requires implementation of social determinants of health applied to a lifetime brain capital strategy. Women’s and children’s health initially need to be addressed to effectively promote brain health for adult women and men. This will be more successfully achieved through global synergistic actions that implement four key components that introduce surveillance, prevention, acute care, and rehabilitation interventions into neurological training applicable to practice and research. Cooperation among all stakeholders regarding clinical decisions can more effectively reduce the global burden of neurological disease. Identification of resources and addressing deficiencies are specific to a community, region or nation (13).

The following proposed fetal-neonatal neurology (FNN) curriculum proposal offers an educational blueprint that can help promote equitable healthcare. Periodic revisions in the educational content requires continual integration of the knowledge gained from neuroscience advances. Necessary educational resources are needed to maintain the operation of these programs. An academic medical center hub model of brain healthcare education can best align to the specific requirements of the community, region, and nation. Improved life-course brain health through career-long learning (14) can be achieved by practice standards extending from this hub out into all spokes of the wheel represented by smaller healthcare facilities. Synergy among all stakeholders promotes clinical practice, educational and research objectives. Application of the 17 sustainable development goals proposed by the United Nations World Health Organization (WHO), with international professional group partnerships can support worldwide efforts to achieve equitable life-course brain health (15, 16).

Interdisciplinary approaches to FNN training strengthen career-long learning for all neurological subspecialties as well as diverse medical disciplines, guided by these WHO sustainable goals (3, 6). Curriculum content should integrate scientific advances regarding knowledge of the neural exposome concept to offer more accurate diagnostic and therapeutic choices. Developmental origins and life course perspectives emphasize critical-sensitive time periods of neuroplasticity beginning during the first 1,000 days. Adjustments will later occur particularly during the person’s adolescence and reproductive senescence. Siloed approaches by stakeholders alternatively impede research, innovation, regulation and funding efforts, resulting in less effective clinical decisions across healthcare disciplines. Interdisciplinary FNN training applied to career-long clinical practice helps promote healthcare, education, and research objectives to benefit all persons through the lens of diversity, equity, and inclusion (DEI). This brain capital strategy applies across neurological subspecialties to reduce mortality and sustain brain health within and across each generation (17).

## Interdisiplinary fetal-neonatal neurology program objectives

Principles-to-practice application of clinical skills in FNN for all neurologists requires an educational curriculum modified for each subspecialty. Adult neurology trainees benefit from a working familiarity of FNN during their time-limited pediatric neurology rotations. An appreciation of developmental disease pathways will later help guide diagnosis and treatment of neurological disorders in the aging brain. Adult neurology residents will be better prepared to consider developmental origins and life-course perspectives during adult practice, education, and research activities relevant to cerebrovascular, neurodegenerative, and epileptic disorders for women and men. Pediatric subspecialty residents, nurses and therapists represent a diverse group of rotating trainees across many disciplines who also benefit from these training experiences. Medical students emersed in FNN rotations during their earlier postgraduate education will strengthen their career paths applicable to the selection of primary care or subspecialty training in their chosen healthcare fields.

Pediatric neurologists who choose FNN as their career path require more intensive bedside and didactic experiences. This training program strengthens their diagnostic skills to be applied when children later require evaluations into young adulthood. More precise identification of life-long neurologic disorders begin with their heightened awareness of risks from early life brain disease exposures over the first 1,000 days. FNN enhances traditional pediatric neurology curriculum training through an educational approach that emphasizes the continuity of reproductive, prenatal, and childhood experiences to better identify risks and consequences from disease and adversity. Emphasis on the woman and the developing maternal-placental-fetal (MPF) triad guide diagnostic perspectives regarding trimester-specific disease stressors that initially may impair the fetal brain. Adverse effects on each person’s dynamic fetal neural exposome begins with prenatal neural maldevelopment expressed as anomalous or destructive lesions that promote neonatal and early childhood neurologic disease consequences.

Prenatal-to-postnatal continuity of risks or injuries contribute to childhood neurologic sequelae. A preterm and full-term newborn minority presents with the “great neonatal neurological syndromes” expressed as encephalopathy, seizures, and stroke (18). These earliest postnatal phenotypes represent disease pathways that often correlate with earlier antepartum time periods and etiologies. Childhood neurologic disorders subsequently present as “the silent majority” who remain asymptomatic during earlier evaluations despite cumulative disease risks or subclinical expressions of disease pathways. Childhood communicable and noncommunicable disease and adversity subsequently are encountered through adolescence that initiate permanent brain injuries, sometimes superimposed on previous vulnerabilities. Childhood TSI subsequently contributes to reduced adult brain health when expressed through reproductive senescence from later life disease and adversity (3).

## Workflow and curriculum content during FNN training

A recommended 2-year FNN program is presented that extends formal training beyond the present one-year programs now offered. Organizations such as the United Council of Neurological Subspecialties (UCNS) in the United States offer certification in neonatal neurocritical care for practitioners who successfully pass an online examination.^1^ Multiple neurological subspecialties previously have received endorsements by the UCNS representing their respective professional organizations. Trainees for all these programs are required to pass an approved certification examination developed by selected experts within each discipline. This process more recently introduced suggested training requirements to satisfy FNN certification. Neonatal neurocritical care (NNCC) is presently emphasized compared with fetal and pediatric learning experiences. Two reasons contribute to this approach: the time restraint that requires accommodation to the currently assigned 12-month training period, as well as the relative paucity of experienced faculty and required resources at most teaching institutions to offer an integrated approach involving the three components of the proposed FNN program. Revisions to the program duration and curriculum content will re-balance fetal, neonatal, and pediatric program components to offer more opportunities to learn specific subtopics during each of the three rotations. These reassessments will require consensus among those designated as the FNN leaders in this growing interdisciplinary field. Re-definition of the minimally competent trainee who can pass the certification exam will require re-consideration as changes in the depth and breadth of the curriculum content are offered during future training.

Improved neonatal neurological care with more systematic pediatric follow-up to clinically manage children with neurological disorders has been the motivation for the organization of these one-year training experiences. Education will presently be achieved primarily through self-administered training, guided by an outline of suggested curriculum topics. Present opportunities to achieve certification by practicing pediatric neurologists and neonatologists will also rely on institution-specific resources to provide protected time to meet FNN program certification requirements. Individual practitioners outside of existing programs alternatively can acquire knowledge of curriculum content based on a case-based learning model. Opportunities to travel and work at more established programs may be possible for selected trainees to strengthen their learning experiences.

The proposal of a two-year program established at an increasing number of combined obstetrical-pediatric medical centers with academic affiliations will more effectively offer comprehensive curriculum directed by experienced faculty. Adjustments in training requirements for trainees will adhere to specific educational objectives guided by mentoring committee recommendations (Appendices 1A–B). Implementation of systematic monitoring of the trainee’s clinical and didactic progress will be part of this educational experience. Protected time beyond patient care will require their respective institutions to provide funding for trainee and faculty salaries requiring the necessary educational materials. These financial obligations may require shared support among the individual institution, government-supported research grant funds and philanthropy.

Interdisciplinary FNN training encompass three integrated neonatal, fetal, and pediatric clinical experiences. A trainee’s time during each rotation will emphasize continuity of care relevant to the first 1,000 days of early brain development. Clinical follow-up through 3 years of age will adjust to the chronologic age of preterm survivors based on their gestational age at birth (Table 1). Throughout this proposed 24-month training period, trainees will participate in supervised “bedside” healthcare encounters, supplemented by didactic “classroom” participation. Both experiences re-enforce curriculum content to be later applied during career-long learning. Knowledge of FNN by all neurologists is best achieved by consideration of a dynamic neural exposome concept throughout the lifespan that preserves brain health.

**Table 1:** Clinical “Bedside” FNN training opportunities.

Clinical settings	Training experiences
Preconception surveillance	Maternal-pediatric diseases/adversities
Obstetrical service	High-risk pregnancies/AMEs
Pediatric service	Childhood diseases/ACEs
First trimester surveillance	Adolescent pregnancies
	Planned vs. unplanned pregnancies
Maternal levels of care	Identify four levels of care based on risk
Sonographic landmarks	MPF triad, placental-cord implantation/development
Serologic screening	Communicable/non-communicable diseases-systemic, neurologic
Genetic screening	Chromosome, microarray, WES, GWAS tests
**Second trimester surveillance**	
Levels of care reassessed	MPF triad health risks identified
Anatomic survey	System-specific relevant to the developing neuroaxis
MFM/genetic referrals	Multisystemic secondary effects on the fetal brain
	Primary brain anomalous/destructive lesions
**Interdisciplinary MFM service**	
Fetal neurology consultations	Serial MPF triad assessments focused on fetal brain effects
Fetal imaging services	Sonographic biometry and brain or systemic anatomical descriptors (e.g., nuchal transparency, brain malformations, general organ anomalies)
	Functional descriptors: state transitions (non-stress test), fetal movements, biophysical scales, doppler flow indices
	Fetal MRI: improved resolution-brain/multiorgan/placental structures
MFM conferences	Serial interdisciplinary diagnosis/prognosis
	Antepartum surveillance/planning for fetal interventions
	Optimal preterm delivery options for mother and child
**Third trimester surveillance**	
	Late pregnancy interdisciplinary evaluations that address worsening MPF triad diseases
	Plan peripartum obstetrical options/exit procedures
**Maternal hospitalizations**	
	Acute infectious, hypertensive, metabolic, mental disorders
	Complications from primary neurological diseases
	Maternal intensive care illnesses
**Peripartum/neonatal services**	
	Obstetrical choices during labor and delivery
	Intrapartum fetal surveillance testing choices
	Acute neonatal neurocritical care practices
	Resuscitation/multisystem priorities by all providers
	Continuity for stepdown neurocritical care
	Multi-system assessments/diagnostic testing choices
	Developmental/therapy/neuropalliative interventions
	Shared decisions/conferences with all stakeholders
	Convalescent stage and discharge planning
	Transitional family conferences with primary care/early Intervention referrals
Pediatric services	
Primary care	Health maintenance/wellness programs/assess ACEs
Neonatal follow-up program	Multisystem/developmental risks/medically vulnerable child
Early intervention program	Multi-therapy interventions/preschool planning
Pediatric subspecialty referrals	
Outpatient clinic evaluations	System-specific including behavior/mental health
Epilepsy service	
Sleep service	
Hospitalizations including pediatric intensive care	Acute presentations/neuropalliative decisions

Modified from Scher (3). AMEs, adverse maternal experiences; ACEs, adverse childhood experiences; WES, whole exosome sequencing; GWAS, genome-wide association studies; MFM, maternal fetal medicine; MPF, maternal-placental-fetal.

## The neonatal neurology component of a FNN program

Neonatal neurocritical care (NNCC) is often required following problematic events during parturition. This will remain the central training focus for trainees to acquire a more comprehensive understanding of this symptomatic neonatal minority who requires intensive care. Resuscitative care paths apply peer-reviewed practice guidelines designed to reduce mortality and morbidity for this vulnerable population. These clinical pathways teach emergent healthcare choices for children who experienced difficult fetal-to-neonatal transitions. Trainees must understand neonatologists’ strategies as they address acute multi-system dysfunction in response to obstetrical practice choices that include emergent decisions. Chosen treatments strive to promote survival and functional recovery. Learning objectives must integrate anticipated healthcare readjustments during acute, subacute, and convalescent stages in the neonatal intensive care unit (NICU) to achieve the most favorable medical status by the child’s discharge. Neurologic phenotypes, however, are often expressions of combined fetal and neonatal disease pathways with increased risks for neurological sequalae. Antepartum injuries to an immature and vulnerable fetal brain more likely contribute to more severe adverse outcomes during parturition that require resuscitation and intensive care. Fetal followed by neonatal complications increase the probability for survivors who later confront life-long neurological disorders beginning during early childhood.

Interdisciplinary neonatal neurocritical care (NNCC) training requires interactions among multiple pediatric medical-surgical subspecialties, including neurological subspecialists. Working knowledge of technical interpretations require patient-specific clinical correlations. This learning process requires selection of appropriate neurophysiological, neuroradiological, perinatal pathological and neurogenetic testing options. Supervised assessments using each testing modality are performed. Trainees need to learn current peer-reviewed classifications regarding neonatal encephalopathy (NE) (19), EEG (20), MRI (21), and placental abnormalities (22) to improve their understanding of disease mechanisms, interventions, and prognosis as they improve their historical and examination skills. Clinical mimicry must be anticipated individually or with different combinations expressed as the “great neonatal neurological syndromes.” This analytic approach will help improve recognize and reduce bias to promote effective shared decisions with families. Diverse disease pathways contribute to abnormal neonatal phenotypes over variable time intervals (3). Cumulative effects of prenatal TSI may previously have threatened MPF triad health resulting in fetal brain injuries. Subsequent clinical expressions during peripartum and neonatal periods may be distant from the onset of the disease pathway.

Educational experiences rely on a multi-systemic approach directed by neonatologists who coordinate multidisciplinary consultations. These educational experiences also require nursing, therapist, child-life, and social work participation. These healthcare providers strengthen the trainee’s bio-social perspectives that promote more accurate clinical skills. Effective developmental care choices with sustained communication efforts with parents and families maintain cooperation and trust. Supervised evaluations with neuropalliative teams further prioritize the importance of effective dialog to support family beliefs and values as they confront their medically fragile children’s challenges (23). These experiences help the trainee learn shared clinical decisions through compassionate care. Learning the science of uncertainty is an essential educational component since prospective diagnostic decisions are provisionally based on limited information (24). Trainees are instructed to pursue greater retrospective understanding of each woman’s reproductive and pregnancy health histories based on additional information acquired at family meetings, supplemented by critical re-review of the medical records of parents, the pregnancy and their child. These insights enhance time-dependent diagnostic choices relevant to MPF triad disease pathways that contribute to neonatal neurological phenotypic presentations. This approach increases awareness of childhood risks for neurologic sequelae and provides a more accurate diagnostic roadmap for providers after discharge.

The phrase “reproductive risk and the continuum of caretaking casualty” was introduced over a half century ago, more recently re-emphasized as the field of FNN emerged (25). Researchers initially designed birth cohort studies to investigate factors responsible for high maternal and pediatric mortalities following World War II (26, 27). Women’s health during their pregnancies in response to TSI was emphasized relative to the prevailing medical care practices during the latter half of the 20th century. Suspected associations with childhood neurologic sequelae such as cerebral palsy and epilepsy in one American-based birth cohort study of mother–child dyads through 8 years of age (28). These early maternal and pediatric research perspectives helped strengthen skills for successive generations of clinicians as the fields of neonatology, maternal-fetal medicine, and pediatric neurology were established and expanded. More demanding training requirements will be required as new information requiring multiple exposome effects are investigated. Diagnostic and therapeutic developments applied to future FNN training will contribute to improved brain health across each lifespan and for future generations.

## The fetal neurology component of a FNN program

Trainees will apply their NNCC learning experiences during multiple types of fetal neurology consultations throughout a woman’s pregnancy. Combined obstetrical-pediatric medical centers provide more comprehensive opportunities to learn reproductive and pregnancy health assisted by multidisciplinary healthcare providers. Supervised fetal neurology consultations during these prenatal clinical encounters strengthen the subsequent neonatal neurology and pediatric components of this proposed FNN program.

Consultations are often initiated based on abnormal fetal surveillance test interpretations. Abdominal sonographic findings are presently the principal source of information that initiate a fetal neurology consultation based on the identification of fetal brain anomalous or destructive lesions (28, 29), often during multi-organ surveys performed between 18 and 22 weeks’ gestation. Accurate neurological assessments by trainees must therefore begin with acquisition of skills to recognize brain lesions (Table 2) initially identified by sonography. Future training opportunities for certification in sonography by fetal neurologists will resemble established programs for fetal cardiology training (30). Interdisciplinary medical-surgical cardiology programs have presented the opportunities and challenges when evaluating children with congenital cardiac lesions to optimize prenatal and postnatal interventions. Similar skills for fetal neurologists will be required by interdisciplinary FNN programs. More effective diagnosis of fetal brain disease pathways associated with anomalous or destructive lesions will select effective neuroprotective interventions. Fetal behavioral assessments representing state transitions help detect abnormal clinical signs using 4-D sonography and doppler studies as important functional assessments (31, 32) when compared with structural abnormalities on fetal neuroimaging. Second and third trimester fetal brain MRI studies often help better detect lesions using the improved sensitivity and specificity of fetal MRI technologies that exceed sonographic resolution. Neonatal brain MRI subsequently offer even more superior postnatal imaging detection. Serial comparisons of prenatal with postnatal sonography and MRI images will improve the detection and evolution of neuropathological lesions associated with fetal brain disease pathways later are influenced by subsequent neonatal followed by childhood diseases.

**Table 2:** Major fetal brain lesions documented by fetal imaging.

Developmental neuroaxis lesion categories	Neural anomaly examples
Neural tube defects	Anencephaly
	Encephalocele/meningocele
	Myelomeningocele
	Caudal regression
Neuronal migration disorders	Schizencephaly
	Lissencephaly
Posterior fossa malformations	Dandy-walker syndrome
	Chiari type II malformation
Ventricular abnormalities	Ventriculomegaly
	Progressive hydrocephalus
Midline brain abnormalities	Holoprosencephaly
	Agenesis of the corpus callosum
	Septo-optic dysplasia spectrum
	Vein of Galen vascular anomaly
Miscellaneous	Hydranencephaly
	Porencephaly
	Tumors
	Hemorrhages

Fetal neurology consultations require knowledge of periconception and early pregnancy disease pathways. More extensive testing choices supplement sonography (33–36) (Table 3). Fetal brain disease pathways may be represented by infectious, serologic, genetic, and xenobiotic biomarkers based on maternal or fetal blood results, together with urine, serous or amniotic fluid analyses. Test interpretations include cell-free biomarkers that improve detection of specific trimester-specific disorders that affect the MPF triad. Accurate FNN diagnostic choices require an understanding of the triad’s physiological responses to TSI, guided by choices and interpretations of diverse testing modalities in addition to fetal sonography selected throughout pregnancy.

**Table 3:** Prenatal diagnostic tests with abnormal outcomes.

Test choices	Maternal-fetal outcomes
Alpha fetoprotein	High: neural tube defects (NTDs), omphalocele, gastroschisis, bowel obstruction
	Low: Down’s syndrome
Combined with free-beta HCG, estriol, Placental-associated plasma protein A (PAPP-A)	Improved detection of NTDs, gastrointestinal anomalies
**Genetic screening tools (parental disease or carrier state)**	
Pre-implantation tests-aneuploidy (parental blood), microarray, WES, GWAS	Chromosomal abnormalities with brain malformations, genetic-metabolic defects-autosomal, X-linked recessive
Early pregnancy-maternal/ fetal cell-free, cell-based Amniocentesis and villus sampling when indicated	Same; mosaicism
**Trophoblastic-based studies (individual, combined)**	
PAPP-A	Ischemic placental syndrome associated with pregnancy-induced hypertension and gestational diabetes; fetal growth restriction; stillborn, prematurity
Vascular endothelial growth factor	Same adverse outcomes
Placental growth factor (PIGF)	Same adverse outcomes
Estrogen (urine, blood)	Same adverse outcomes
Placental lactogen	Same adverse outcomes
Uric acid	Same adverse outcomes
**Later pregnancy biomarkers**	
Biometry and anatomical descriptors	Fetal growth restriction, anomalies, destructive lesions
**Biophysical scales (>32 weeks GA)**	
Nonreactive stress test (± vibroacoustic stimulation)	Altered arousal states, dysautonomia
Fetal breathing movements	Dysautonomia vs. primary pulmonary disease
Fetal muscle tone	Low and high tone states
Amniotic fluid volume	Polyhydramnios, oligohydramnios
Fetal movement count reportage	Loss of fetal well being
Pulsatile umbilical/meningeal artery indices	Loss of fetal well-being, fetal brain disorders or stillbirth
Toxicology screening	Effects of neonatal abstinence syndrome (associations with maternal mental health disorders, socioeconomic stresses and lifestyle choice)

Early pregnancy biomarkers often represent trophoblastic maldevelopment associated with diverse uteroplacental disease pathways that began days after fertilization. Placental, cord and uterine pathologies often interact to produce adverse outcomes expressed by the MPF triad. This often follows first trimester developmental consequences affecting the remainder of the pregnancy (18). Three principal placental disease mechanisms need to be individually considered or in different combinations to interpret test results to accurately represent complicated disease effects on the MPF triad.

Maternal immune activation (MIA) (37), ischemic placental syndrome (IPS) (38) and fetal inflammatory response (FIR) (39) individually or through different combinations contribute to diverse MPF triad diseases which contribute to fetal brain injuries. MIA represents varying degrees of early pregnancy graft vs. host immunological incompatibility that may contribute to fetal death with a miscarriage. Survivors alternatively experience impaired placental nutritional and growth substance delivery and waste removal. Fetal progenitor neuronal population abnormalities may result that subsequently promote maladaptive consequences with suboptimal fetal brain connectivity with continued maturation. Two histopathological lesions exemplify T-cell mediated incompatibility associated with MIA, villitis of unknown etiology (40) and maternal floor infarction/massive perivillous fibrin deposition (41). Another disease pathway associated with FIR also resembles early pregnancy immunological intolerance like MIA (39). Pathogen-associated communicable diseases as well as non-communicable immune incompatibility disease pathways need to be considered when formulating a differential diagnosis.

IPS is associated with defective angiogenesis within the functional hemochorial placenta as the secondary yolk sac is replaced following 10–12 weeks of gestation. Maternal or fetal malperfusion lesions associated with IPS can contribute to destructive fetal brain injuries. Greater risks emerge as fetal growth demands increase throughout the latter half of pregnancy. Etiopathogenesis involve disease pathways that begin with first trimester contributions. Abnormal precursor trophoblastic development will lead to impaired placental vasculature associated with maternal decidual or fetal villous circulation. Resultant brain injuries may be combined with pre-existing MIA-induced abnormal fetal brain changes (38, 42).

FIR represents two forms of inflammatory responses that are experienced during early or late pregnancy (39). The earlier disease pathway was described above leading to miscarriage as an MIA-related brain disease pathway involving immunological incompatibility. Later presentations of FIR often result from ascending vaginal infections that invade the placental membranes. The evolution of this disease process influences the initiation of parturition, resulting in preterm or full-term suboptimal deliveries associated with fetal brain injuries. Etiopathogenesis associated with FIR-related brain injuries encompasses combined hypoxia-ischemia and inflammatory-mediator adverse effects on neuronal structure and function, expressed across defective placental and fetal blood–brain barriers (43). Earlier first trimester cord anomalies and uterine myometrial lesions also contribute to increased risks for adverse outcomes when combined with MIA, IPS or FIR effects throughout the pregnancy.

Knowledge of reproductive and early MPF triad conditions strengthens the trainee’s appreciation of cumulative adverse effects on early fetal brain structure and function following fertilization and early placentation. Maldevelopment of transient structures such as within the ganglionic eminence or subventricular and subplate zones during the first half of pregnancy contribute to later adverse effects as cortical and subcortical structures develop during the second half of pregnancy (3). A comprehensive etiopathogenetic approach must consider interrelated MPF triad disease pathways that are continually influenced by TSI over three trimesters. Maternal communicable and non-communicable illness and adversity contribute to adverse prenatal exposome effects expressed as fetal brain injuries. An understanding of fetal multisystemic disease effects is essential resulting in secondary fetal brain injuries, exemplified by cardiac, pulmonary, gastrointestinal, and renal disorders. Greater details of these topics have been discussed elsewhere (3).

Knowledge of four presently defined levels of maternal care (44) provide trainees with an understanding of current practicalities regarding choices of obstetrical practice that identify and respond to pregnancy risks. Each woman and her partner’s reproductive health prior to each pregnancy must be considered when selecting an appropriate level of care. Suggested improvements by stratification of risk are being investigated by the assignment of risk-appropriate (45) choices. Parental childhood diseases and adversities may impair fertilization before conception. Early placentation abnormalities during the first trimester contribute to embryonic and fetal brain disorders. These risks need to be anticipated particularly with unplanned pregnancies, whether in high income or low-to-moderate resource countries (46). Adolescents of reproductive age represent one vulnerable population of young women who more often require high-risk prenatal care (47). Preconception testing helps preserve or improve early pregnancy health for women of all ages, particularly for couples seeking medical interventions for infertility. More advanced diagnostic options will help anticipate risks leading to fetal brain injuries from MPF triad diseases detected throughout the first half of pregnancy.

Second and third trimester fetal neurology consultations subsequently may be required when complications are more definitively identified. Negative effects on MPF triad health often present closer to delivery which can impair parturition physiology (48). Time-sensitive diseases during the latter half of pregnancy potentially contribute to destructive fetal brain lesions. Systemic maternal illnesses are associated with fetal brain injuries including hypertensive, diabetic, infectious, autoimmune, and mental health disorders. Adverse outcomes may occur despite reasonably effective medical interventions in response to observable clinical signs, offering limited diagnostic and therapeutic options (3, 18). Women’s primary neurological disorders such as intellectual disabilities, epilepsy, multiple sclerosis, and neuromuscular disease (49) introduce greater risks, particularly when combined with multisystemic disorders experienced during pregnancy, such as hypertension and diabetes. Complicated MPF triad conditions more often involve multiple clinical pathways that cumulatively contribute to increased risks for women with adverse effects to their children resulting in fetal brain injuries.

Clinical illnesses experienced during the latter half of pregnancy will benefit from interdisciplinary genetic, fetal imaging and multi-disciplinary maternal-fetal medicine discussions. While fetal neurology consultations may not be formally requested, FNN training assists fetal neurologists when they participate in obstetrical and neonatal interdisciplinary discussions and conferences. Neurological perspectives can influence pregnancy management strategies and prognostic predictions particularly with complex medical situations. Trainees gain important insights regarding prenatal medical or surgical intervention choices specific to the available medical services provided at a particular obstetrical-pediatric medical center. Specific fetal medical-surgical interventions, vaginal vs. caesarian delivery options, and exit procedures may need to be considered. Fetal transfusion treatments, fetal neurosurgical closure of dorsal neural tube defects and removal of a cystic hygroma exemplify procedures that are offered during complicated pregnancies at quaternary obstetrical-pediatric centers. Innovative rescue neuroprotective procedures continue to be investigated which will emphasize the importance of preventive rescue, and reparative treatment options prior to the child’s birth.

The FNN trainee’s participation during maternal hospital rounds offers important perspectives when critically ill women require hospitalization to treat acute diseases. Increased risks for fetal brain injuries can result when women are confronted with significant medical complications. Severe preeclampsia, diabetic ketoacidosis, ascending genitourinary infections, sepsis, and acute mental health crises are principal reasons for these hospitalizations. This cohort of women also is more likely to experience postpartum complications which also increase their risks for death. This outcome is comparatively more likely with women who confront healthcare disparities (12). Medical errors of omission or commission regarding diagnosis and treatment options can result when multiple sources of bias are applied by providers, such as implicit, selection, and premature closure biases. These treatment challenges are particularly experienced by women and children who experience health disparities (50).

### Peripartum considerations after fetal neurology consultations

Peripartum assessments of fetal distress during dysfunctional parturition remain essential to the fetal neurological component of FNN training. Clinical interpretations of fetal heart rate, oximetry, and intrauterine pressure monitoring results help qualitatively identify loss of fetal well-being sometimes associated with increased risks for fetal brain injuries. An understanding of the neuroprotective role of the robust evolutionary peripheral chemoreflex response is required which helps the trainee recognize why most fetal brain injuries are less severe or can be avoided (51, 52).

Trainees require an understanding of the obstetrical team’s prospective choice of maneuvers when attempting to preserve the child’s and mother’s health, by recognizing the science of uncertainty (25). Time-dependent decisions are often executed without prior knowledge regarding the presence or extent of antepartum MPF triad diseases given testing limitations and lack of historical details. These diagnostic obstacles are comparatively more likely in lower-level hospital care settings from which transport to level three facilities for the mother or neonate were required.

Adverse outcomes may also result in response to a woman’s request for specific medical interventions to deliver her child, preferably by vaginal delivery procedures. Complications may occur based on undetected antepartum uteroplacental vulnerabilities such as defective placental implantation as well as cord (53) or myometrial abnormalities such as adenomyosis (54).

Partnership among obstetrical team members include midwives and doulas who can improve pregnancy literacy and promote more positive experiences for women and their partners. Respect for racial, religious, and cultural differences need to be recognized. These modes of prenatal healthcare delivery are particularly valuable in LMIC and HIC healthcare deserts where more limited resources prevail (55).

Neuroprotective protocols may require emergent resuscitative measures for children who expressed signs of fetal distress followed by neonatal encephalopathic signs (NE). Abnormal parturition associated with or without active labor may be associated with fetal heart rate abnormalities identified by three accepted abnormal pattern categories. Degrees of fetal distress as defined by these abnormalities offer guidance for alternate obstetrical options. However, there are no reliable associations that accurately predict the onset or worsening of brain injury based on current peer-reviewed research (56, 57). Unanticipated abnormal events may occur with or without more easily recognized sentinel events. Inborn neonates more likely will receive immediate access to neonatal resuscitation requiring transfer to the NICU. Outborn neonates are transported after resuscitation to higher level facilities that delay more effective intensive care interventions. Obstetrical and neonatal teams may also provide coordinated emergent medical interventions without knowledge of comprehensive reproductive and pregnancy histories during the peripartum time interval when emergent clinical decisions must be chosen.

Therapeutic hypothermia (THT) (58, 59) has been offered after specific clinical signs of NE are identified and accepted criteria met (60). This intervention is often offered when significant metabolic acidosis is determined on cord blood gas analysis. Limited improvements have been reported based on specific developmental domain outcomes by preschool ages of children. More severe clinical presentations, equipment unavailability and delayed presentation are reported that reduce the effectiveness of THT (61). No definitive outcome improvements have been reported based on current research results that combine THT with erythropoietin (62). Sarnat class 1 presentations of NE are also comparatively more problematic. Unanswered questions regarding treatment efficacy are based on different timing and/or etiological scenarios. Encephalopathic neonates may not have incurred contemporaneous with brain injuries that require or benefit from THT intervention. The proposed time window during which acute disease may be responsive to THT is within 4–6 h, during which reduction of more severe programmed neuronal death can be achieved following hypoxia-ischemia. Complex etiologies over a longer time course may contribute to worse outcome from disease pathways (63, 64). Postnatal placental histopathological analyses may implicate antepartum disease pathways associated with MIA, IPS and FIR as previously discussed. THT administration after non-modifiable diseases such as FIR have no evidence-based benefits to provide fetal or neonatal brain rescue. Alternate therapeutic protocols require new research investigations that will address multiple disease pathways associated with neuroinflammation (65, 66).

Future studies require protocols that will consider timing and etiologies that influence MPF triad health. Reports of positive outcomes remain currently limited, given heterogenous subject selection without accurate stratification of risk. Post-hoc analyses of placental pathology are needed in future research protocols to help identify increased risks for sequelae. Four categories of placental lesions established by a panel of international experts in perinatal pathology have been presented and discussed (67, 68). Cord anomalies often accompany these placental lesions and are also associated with neurologic sequelae. Abnormal length, coiling, knots and insertion often originate during placentation in the first trimester (69). These retrospective analyses of placental-cord diseases will have increased prognostic significance when compared with single or serial neuroimaging interpretation. Cranial sonographic followed by postnatal brain MRI findings compared with fetal neuroimaging will more definitively offer etiologic considerations over different time intervals (70). Novel antepartum biological-chemical biomarkers will combine “omics” technologies with fetal neuroimaging findings to better identify abnormal molecular effects on the neural exposome from TSI before and during the peripartum period (3). Accurate detection of preclinical MPF triad diseases will more accurately identify dyads who will experience dysfunctional parturition and potentially benefit from current neuroprotective rescue interventions such as THT and erythropoietin. Proposed use of reparative neuroprotective therapeutics will apply innovative neuroscience investigations to identify biomarkers associated with chronic stages of neurodegeneration following weeks to months after hypoxic–ischemic onset (71). Serial use of fetal neurological testing will better detect and follow disease progression prior to the peripartum and offer future effective prenatal and postnatal neuroprotective interventions.

## The pediatric component of a FNN program

FNN training continues following the child’s discharge from the NICU, comprised of serial pediatric evaluations in the outpatient and inpatient clinical settings. Coordination among primary care, hospital-based neonatal neurodevelopmental follow-up, and community early intervention programs provide opportunities to assess children to better document normal or abnormal neurologic phenotypes during their first 3 years. This early life support will subsequently be applied to interventions into the child’s school years.

Children with prenatal and neonatal diseases more likely will present during the first 3 years with primarily developmental, epileptic and non-epileptic waking or nocturnal paroxysmal disorders. Other children may remain comparatively more resilient with less expression of neurological signs during early life. Positive functional neurodiversity (72) with less severe or no neurologic disease expression at younger ages may therefore be reassuring. However, early age-appropriate neurologic functions alternatively may be misleading. Pervasive behavioral and mental health disorders might later present at older ages. Positive adaptive or negative maladaptive childhood outcomes through adolescence will depend on person-specific G x E interactions, supported by the family with assistance from interventional services. This strategy is based on the continuity of prenatal to postnatal disease and adversity exposures that encompass TSI with expression throughout childhood and adolescence.

While universal surveillance of neurodevelopmental status of survivors is now being reported by a Children’s Hospital Neonatal Consortium, commonalities and variances in practice highlight the continued need for future data sharing to develop best practices that will strengthen pediatric outcomes (73). Preventive wellness approaches provide trainees with primary pediatric and family-based care perspectives based on office and bedside clinical skills. Identification of adverse childhood experiences contribute to negative outcomes that encompass social determinants of health, substandard parenting skills and poor lifestyle choices. Increased vulnerabilities are particularly experienced by individuals who have also been confronted with medical complications (74). Generalizability of these findings to non-academic and smaller follow-up programs remain an ever-present challenge. This cited consortium report did not address details of the woman’s reproductive health or MPF triad health during pregnancy that influence the child’s early neurodevelopmental status. School-age cognitive disorders such as executive dysfunction or attention deficit disorder as well the DSM-5 defined mental health disorders such as anxiety and depression may later appear. Low birth weight or extremely preterm survivors (75) will require particular attention.

Interdisciplinary exposures by FNN trainees to developmental neuroscience requires an understanding of the translational relevance of animal to human comparative models (76). Developmental rehabilitation science also impacts the child’s motor, communicative and adaptive skills. Enriched environments can be offered by parent-mediated healthcare together with professional developmental interventions. Brain computer interventions based on new technologies will optimize the abilities of children who are challenged with vision, auditory, neuromotor, communicative or neurocognitive deficits.

### Importance of population-based registries

The WHO-ICF disability model has been an important component of community care disability optimization models since it was adopted by international consensus in 2001 (77–79). This concept supports a research component involving collaborative registries that develop and apply biomarkers to detect early-onset neurodevelopmental disorders. Commonalities regarding disease pathways regarding these registries will include categories such as children with fetal malformations, neonatal encephalopathy, extreme prematurity, genetic disorders, early onset epilepsy, and global developmental delays. Community registries are essential to support partnerships between public health agencies and special education systems that maximize needed resources to a region or nation. These registries will strengthen shared decision-making as children with diverse neurologic phenotypes are identified during the first 1,000 days such as autism spectrum, intellectual disability and communication disorders. A life-course strategy can subsequently be chosen that optimize brain health for survivors who express these phenotypes across the lifespan. Informational and genomics science when appropriately applied using deep-state learning can assist with these population-based registries to improve diagnosis and neurotherapeutic choices (80).

Public health agencies for children with special health care needs require population-based registries that examine physical, neurodevelopmental, and family health outcomes during the first 5 years of life with kindergarten entry. Emphasis on early identification, prevention and optimization through informed educational neuroscience practices (81–84) begin with school entry 5–6 years of age based on preschool neurodevelopmental assessments supported by public health initiatives (85–88). These registries must provide equity by offering interventions that address social determinants of health. Application of exposome measures to detect and follow TSI over developmental time include structural and functional gene–environment biomarkers that represent reproductive, pregnancy and early life disease and adversity through age 3 years.

Cognitive and mental health disorders may be expressed at older ages after neurodevelopmental surveillance is completed with termination of these FNN programs by the child’s third birthday. Primary pediatric care or pediatric emergency care interventions subsequently represent the principal healthcare pathways when neurologic disorders later present. Prediction of later childhood challenges will be particularly difficult for those symptomatic younger children who “outgrow” earlier developmental delays or never clinically express early-life developmental disorders such as cerebral palsy, language delay or autistic spectrum disorder. Long delays often separate the onset of fetal or neonatal disease pathways resulting in pervasive dysmaturation or injury (89), and later clinical presentations during childhood, adolescence or early adulthood (90).

### Continuity of neurologic care into later childhood and adolescence

Outpatient and hospital-based pediatric neurology consultations provide additional interactions with medical-surgical subspecialities when communicable and non-communicable system-specific diseases are identified throughout childhood. Pediatric neurointensive care consultations address the most critically ill subset of children who often involve medically fragile neonatal survivors. These populations require enhanced attention to address resource needs given increased risks for reduced life expectancy before adolescence or early adulthood. Recent meta-analyses present pooled data regarding survival primarily for adult cohorts (91). These algorithms predict earlier death during adult years, inclusive of those who experienced developmental disorders with achievement of ambulation and more functional abilities. Only 4 out of 70 published cohorts studied represented a younger pediatric population who were comparatively at higher neurologic risk for childhood death. Inclusion of mortality prediction for these more medically fragile childhood survivors need to be addressed to assess their risks for earlier death before adulthood. This approach realistically includes estimates of reduced life expectancy for those children who experienced early life severe brain disorders.

Supervised clinical encounters through 3 years of age in this proposed FNN program can nonetheless improve the trainees’ abilities to consider pre-school developmental interventions for educational needs. This chosen age limit adjusts to preterm survivors who require chronologic age correction based on their younger developmental ages to compensate for their gestational immaturity at birth. Serial assessments will help anticipate future behavioral and psychometric testing for older preschool and school-aged children. Cognitive, behavioral, and mental health challenges for older children will later require more standardized evaluations using neuropsychometric testing instruments that adjust for gender, race, socioeconomic class, and ethnicity. FNN trainees require a working knowledge of these testing tools. Equitable educational opportunities must be offered to children with developmental disorders living in low-and middle-income countries (LMIC) as well as healthcare deserts within high-income countries (HIC) (92). FNN trainees must integrate DEI principles into shared clinical decisions to alert all stakeholders to provide medical and educational interventions for all children.

## Didactic experiences for the FNN trainee

Non-clinical didactic opportunities for FNN trainees involve “classroom” teaching objectives that re-enforce clinical experiences gained with patient encounters. These experiences will re-enforce the perspectives of brain health or disease across prenatal, neonatal, and pediatric time intervals during the first 1,000 days of brain development that influences life-course brain health. Learning objectives are monitored by a diverse choice of faculty who offer discipline-specific expertise to each trainee while placing sustained emphasis on the importance of DEI. Potential teaching faculty include multiple pediatric subspecialists, as well as nursing, engineering, computer science and bioethics representation. Trainees’ early selection of their mentoring committees should include participants who will support their individualized career-long objectives that sustain professional growth. Research preparations, academic advancements and health care policy advocacy should be encouraged as specific career objectives are established.

All trainees over periodic intervals require evaluations to monitor their developing skills by adhering to current graduate medical education standards for inpatient and outpatient healthcare. A final certification examination as previously discussed is currently offered by an American-based organization identified as the United Council of Neurological Subspecialties (UCNS). Two-hundred representative test questions were initially prepared to test information acquired based on current FNN curriculum content. Practicing clinicians who qualify to take this first version of the exam must rely on their clinical practice experiences at their respective medical centers. They may have limited or no formal training experiences from experienced teachers or mentors. Test questions currently emphasize NNCC topics with comparatively less emphasis on fetal and pediatric components of this FNN program. Practical choices for determining the pass-fail set point for each exam question adjusts to the abilities of the minimally competent trainee as defined by the Angoff method (93). This certification process must adjust to healthcare disparities in LMIC including the Global South (94). Revised questions will need to prepare future test questions that more comprehensively represent an integrated approach involving the three components of this proposed FNN training program (Appendix 2). Emphasis on reproductive and pregnancy TSI effects on fetal brain health will stress the dynamic fetal neural exposome that will influence neonatal, pediatric, and adult brain health. These educational goals can be more realistically achieved over the proposed two-year training time for trainees who are then required to pass this revised certification examination.

Regularly scheduled lectures will be led by trainees, supplemented by informal small group discussions with faculty supervision. Resident and medical student participation will further enrich these interactions. Departmental grand rounds and visiting lecturers help introduce trainees to a wider range of FNN topics. Involvement by multiple residency directors across disciplines, particularly in pediatrics, neurology, obstetrics, and psychiatry will help expand curriculum development through their respective educational perspectives.

Educational science principles need to be maintained to improve neuroscience teaching techniques directed to trainees in existing FNN programs as well as to additional sites when established. Modifications in teaching skills for rotating residents and students need to adjust to their less extensive training opportunities and requirements. Diagnostic skills specifically needed for a specific clinical situation will be adjusted to the full-time trainee, rotating resident or student (81). Teaching strategies will also be adjusted to educational objectives required for students earlier during their post graduate training during medical school rotations in pediatric, obstetrics and neurology (95). Recognition of the heterogeneity of available educational assessment tools for trainees require modifications based on regional and national resource availability specific to HIC or LMIC. Evidence-based educational practices require appropriate pedagogical approaches adjusted to the trainee’s learning environment either at a major teaching center or smaller community-based program. Access to computer-assisted teaching equipment can enhance artificial intelligence assisted learning, provided that careful oversight by teaching faculty is offered. Healthcare policies that prioritize DEI (96) need to emphasize women’s reproductive and pregnancy health for each trainee.

Planned research projects for the trainee will consist of representative case reports with literature reviews, systematic reviews, or primary research projects. Presentations at academic meetings and membership in professional organizations as junior members are to be encouraged. Submission of at least one manuscript for peer-reviewed consideration will be the mentoring committee’s expectation. Preparations for doctoral defense or for training components of NIH-supported research grants will adhere to the aims and objectives specific to these educational experiences for the specific trainee.

Combined clinical and didactic learning experiences provide trainees enhanced opportunities to recognize time-dependent fetal and neonatal diagnoses. This two-step diagnostic process requires acquisition of the necessary clinical skills to serially recognize evolving neurologic phenotypes while concurrently acquiring knowledge of disease pathways that consider genetic through multi-system interactions (18). Learning developmental neuroscience principles require critical review of the peer-reviewed scientific literature cognizant to review if continual updates. Cognitive approaches to decision-making must prioritize bio-ethical and bio-social principles that foster more effective communication among all stakeholders (97). Emphasis on DEI regarding maternal-pediatric healthcare issues address healthcare disparities that negatively affect women and children. More adverse outcomes for adult women and men may later be expressed across the lifespan. Recognition of healthcare deserts in HIC and LMIC-based communities are essential (98, 99) to address maternal and pediatric disparities that increase risks for adverse life-course outcomes. Improved neuroprotective strategies need to expand beyond the white male standard that traditionally dominated past research study objectives. Models of healthcare and disease prevention and treatment must consider women’s healthcare perspectives anchored by DEI principles (100).

Table 4 summarizes the objectives for this proposed interdisciplinary FNN program. Clinical practice, educational and research goals are individualized for trainees with the assistance of their mentoring committees. Teaching faculty in FNN will continue to strengthen their skills by continued involvement with FNN practice. Their educational contributions will need to adjust to the specific teaching center as their knowledge base strengthens through career-long learning. Public health advocacy for women’s and children’s healthcare must remain a high priority, guided by DEI for both trainees and faculty. Established programs will require re-validation of curriculum content to meet certification based on future scientific advances in FNN-related topics. Curriculum revisions will be incorporated into evidenced-based educational teaching tools when preparing updated certification examination questions.

**Table 4:** Objectives for a fetal neonatal neurology program.

Improve clinical/educational pathways for trainees and teaching faculty
time-specific: reproductive health, three trimesters of pregnancy, early childhood
etiology-specific systems biology to recognize age-dependent phenotypes such as cerebral palsy
gene–environment interactions affecting reproductive and pregnancy health based on toxic stressor interplay
Consider disease pathways that represent maternal-placental fetal triad, neonatal and childhood phenotypes
Trimester effects from maternal immune activation, ischemic placental syndrome and fetal inflammatory response
“The great neonatal neurological syndromes”—preterm-full term encephalopathy, seizures, stroke
Developmental disorders and paroxysmal disorders during the first 2 years
Ontogenetic adaptation process redefines ontogenetic adaptation or maladaptive neuroplasticity expression
Informatics and computer science infrastructure
Integrate demographic, clinical, neurophysiologic, imaging, pathology, and genetic metadata sets
Support a relational database with statistical/epidemiologic expertise
Create/expand research programs with NIH-NSF-philanthropic support
Institutional interdisciplinary collaborations
Within departments/across schools (e.g., Medicine, Engineering, Nursing)
Multi-institutional collaborations-birth cohort, cross-sectional, longitudinal research designs
Collaborations with professional societies-white papers, guidelines etc.
Public health policy advocacy (regional, national, international) through governmental, UN-WHO, NGOs regarding DEI

## Life-course effects to a dynamic neural exposome

FNN curriculum introduces the trainee to an understanding of exposome effects affecting the woman, MPF triad, fetus and young child through 3 years of age. Serial evaluations provide early life perspectives when assessing clinical phenotypes that represent a dynamic neural exposome (3). Prenatal and early postnatal consequences often contribute to permanent effects during these first 1,000 days of brain development, recognized as the first critical/sensitive period of neuroplasticity. Preclinical disease onset with evolution often precede phenotypic expressions over variable time intervals with both short-term adverse outcomes throughout childhood and long-term adverse outcomes during adulthood. Disease pathways often remain difficult to detect based on current examination and testing sensitivity and specificity limitations. Therefore, perspectives that apply FNN curriculum content must remain relevant to neurology principles and practice across the lifespan.

The concluding sections re-enforce the importance of formal FNN training for all neurological subspecialties to strengthen career-long learning. Accurate diagnoses and interventions can be offered that will maintain brain health. Cumulative clinical encounters are considered in the context of new peer-reviewed research advances that apply evaluations of TSI effects to a dynamic neural exposome. Unique biological-chemical interactions expressed by each individual involve tens of thousands of xenobiotic exposures over one’s lifetime (101). Variable resistance or vulnerability define the presence and extent of neurologic disorders given person-specific G × E interactions that influence neuronal mechanisms (102). Adverse geopolitical and climate conditions further increase the complexity of risks for sequelae (103).

Development of untargeted and targeted quantitative screening tools will enhance diagnostic and therapeutic options regarding complex interactions of factors that negatively alter neuronal connectivity. Metadata informatic analyses using artificial intelligence (104) will utilize deep-learning strategies to enhance assessments of reproductive and pregnancy health, exemplified by advances in the field of artificial reproductive technologies (71). More accurate biomarkers are needed to detect trimester-specific disease states of the MPF triad to select more precise therapeutic interventions. Adherence to DEI principles with each clinical encounter re-enforce for health providers in LMIC and HIC medical deserts relevance to improve diagnostic acumen (105).

### Reproductive and pregnancy effects

Reproductive health risks of women and their partners begin before each pregnancy which subsequently diminish MPF triad health that contribute to fetal brain lesions. Girls of reproductive age and women who experienced childhood neurologic diseases threaten their health during pregnancy. Developmental disorders, epilepsy, multiple sclerosis, neuromuscular disease, and mental health disorders exemplify such disorders (49). Childhood systemic diseases contribute secondary detrimental effects on the fetal nervous system during pregnancy such as diabetes, hypertension, obesity, autoimmune disorders, mental health disorders and genitourinary/congenital infections (3). The recently defined field of matrescence (106) emphasizes positive and negative maternal effects during each pregnancy that influence women and their children, even when experienced after reproductive senescence. Maladaptive effects during each pregnancy impair the MPF triad through inflammatory, oxidative, and hypoxic disease pathways. Greater susceptibility to trimester-specific infectious, thrombotic, and hemorrhagic maternal disease states consequently contribute to fetal brain anomalous or destructive lesions (107). Risks are worsened by health care disparities confronted by vulnerable populations.

Genetic vulnerabilities from inherited parental and familial risks may be introduced during mitosis after conception. Complex post-translational modifications subsequently alter the developing fetal brain which may activate disease pathways by mechanisms, such as imprinting, somatic mosaicism, and epigenetic alterations (e.g., methylation, phosphorylation, histone modification etc.). G × E interactions continue to amplify these TSI effects as pregnancy continues which further reduces MPF triad health. Novel biomarkers will apply genomics research technologies to offer informational platforms that more precisely identify phenotype–genotype comparisons to more precisely select therapeutic options (108).

Positive or negative exposome effects on the MPF triad represent G × E interactions based on the placental exposome (3). MIA, IPS and FIR, as discussed in the previous section, represent three interrelated trimester-specific placental disease pathways that adversely influence the fetal neural exposome. These placental diseases interact to impair MPF triad functions which contribute to fetal brain lesions. Significant adverse brain health effects have been reported based on identified placental gene-trait associations (109). Distal mediator-enriched transcriptome-wide placental biomarkers have been reported for a cohort of an extremely preterm survivors. These measures represent placental genomic dysregulation that result in altered developmental fetal brain programming expressed as specific short and long-term outcome diseases. Larger populations will more comprehensively identify additional genomic traits for a wider selection of neurologic sequelae.

Etiopathogenesis begins at conception based on parental reproductive risks which introduce genetic defects into both the embryonic placenta and fertilized oocyte following mitosis. Abnormal genetic codes contribute to placental maldevelopment associated with early precursor trophoblastic functions at the embryonic decidual-fetal interface beginning 2–8 days following conception. Abnormal development of the hemochorial placenta after 10 weeks post conception later impairs angiogenesis and villous maturation through the third trimester. Progenitor neuronal populations within the embryonic fetal brain form within the neural plate and crest beginning at 17–19 days post-fertilization. Similar abnormal genetic codes also contribute to dysmature development of microglia, macroglia or interneuron populations which negatively influence region-specific axonal and synaptic connections (3). This abnormal fetal neural connectome may result from MPF triad diseases that progress throughout a preterm or full-term pregnancy. Proposed volumetric and functional brain-placenta MRI imaging comparisons will more accurately detect suboptimal interactions in response to TSI (110, 111). Cumulative negative effects to this dynamic fetal neural exposome will subsequently contribute to suboptimal responses during parturition for those who then require neonatal intensive care. Preterm and full-term expressions of the “great neonatal neurological syndromes” of encephalopathy, seizures and stroke will subsequently require NNCC interventions.

### Childhood and adult effects

Antepartum, peripartum and neonatal disease pathways cumulatively increase risks for brain disorders following NICU discharge. Childhood communicable and noncommunicable diseases experienced at progressively older ages initiate or worsen brain injuries. Risks for neurologic sequelae increase following infections, cranio-cerebral trauma, or multisystemic diseases which separately or collectively impair the childhood nervous system with adulthood effects. Adverse childhood experiences related to social determinants of health, suboptimal parenting skills, poor lifestyle choices and xenobiotic exposures further enhance risks for neurologic morbidities. Childhood neurologic dysfunction may be expressed as any combination of motor, cognitive, language and social adaptive skill deficits. Developmental disorders and epilepsies are earlier neurologic sequelae expressed over the first 1,000 days which represent the concept of developmental origins of later disease expression (112). Cognitive, behavioral, and mental health disorders subsequently appear through older school ages into adolescence based in part on early life diseases (113).

Adults remain susceptible to neurologic disorders throughout the aging process. Adult illnesses combined with adverse life experiences represent later life expressions of brain diseases based on early life experiences (74). Infections, trauma, and multi-systemic diseases contribute to injuries in the aging brain. Compelling evidence has been presented that correlate developmental origins of disease with adult-onset neurodegenerative disease expression (114), exemplified by dementia (115), Parkinson-like disease spectra (116, 117) and neuroimmunological diseases (118–120). Anticipation of these adverse outcomes can be more effectively reduced using preventive (121) supplemented by rescue and reparative interventions. Nutritional improvements, reduced xenobiotic exposures, sustained exercise regimens and time-sensitive mental health therapies each or collectively contribute to positive effects during later life. Greater education regarding women’s and children’s health are essential to promote adult brain health. Equitable public healthcare policies must be sustained throughout the lifespan beginning before each pregnancy. Continuity of life-course brain health must first advocate for the health and well-being of women during each pregnancy that will benefit multiple generations of children maturing into adulthood. This essential life-course perspective requires investment in a brain capital strategy which can be strengthened by interdisciplinary FNN training for multiple neurologic subspecialties (17).

## FNN training supports neurotherapeutic research advances

Interdisciplinary FNN training will help advance collaborative efforts to design more effective preventive, rescue, and reparative neuroprotective protocols. These research efforts will contribute to a life-course brain capital strategy by choosing etiology-specific interventions as a function of time. Exosome research exemplifies one such research effort. Tissue-specific biomarkers are being developed that can be designed to detect MPF triad, neonatal or childhood disease pathways (110, 111). Diagnostic choices during critical-sensitive time periods of neuroplasticity begin during prenatal life. Placental and fetal neuronal-derived exosomes are currently being investigated that both detect disease and deliver neuropharmacological agents using placental transport systems. Drug selection will target developmental disease pathways that impair vulnerable brain structures specific to the developmental stage during the pregnancy within the embryonic and fetal neuroaxis (122, 123).

Disease states and therapeutic responses can also be more closely monitored using serial quantitative fetal neuroimaging tools (124, 125). Quantitative fetal brain MRI measures now document altered measures that represent underdeveloped or impaired neuronal connectivity. Such abnormalities are more likely to occur during the first 1,000 days, particularly for those mother–child dyads exposed to socio-economic adversities in poorer neighborhoods (126). Brain health risks begin with the identification of factors that impair a woman’s health before and during each pregnancy with consequential reduced childhood neurologic health over the first 2 years of life (127). Neuroimaging abnormalities later during adolescence (128) represent socioeconomic contributions to longer term negative consequences into late childhood. These same brain structure technologies will contribute to the design of longitudinal studies that will assess developmental origins of health and disease across the lifespan (121).

## A brain health strategy requires equitable healthcare delivery

Interdisciplinary FNN curriculum training strongly supports maternal-child public health initiatives to promote life-course equitable health care. Early life reproductive and pregnancy health effects on the MPF triad will have profound later life consequences. Siloed brain research, innovation, regulatory, and funding systems presently fall short of reaching a more comprehensive understanding of the development-aging continuum that addresses brain health for all persons. A life-course brain capital strategy will offer interventions to reduce or avoid neurologic disorders into old age and across generations by addressing health disparities. Emphasis on the science of biological-to-social transitions need to recognize the social determinants of health to reduce adverse effects through diversity, equity and inclusion. Interdisciplinary FNN curriculum can help promote multi-institutional research collaborations to design such studies to more accurately guide person-specific diagnostic and therapeutic recommendations. Future studies must identify biomarkers of adaptive or maladaptive neuroplasticity that represent a dynamic neural exposome in the context of brain maturation and aging.

The United Nations Agenda for over the last quarter century has advocated for 17 sustainable development goals (SDGs) to address the global burden of disease (129). These goals can assist diverse populations dependent on the necessary resources needed to overcome disparities. Limitations exist in both LMIC and HIC medical deserts that increase the global burden of neurological disease (130). Greater attention to the adverse long-term effects following prenatal and childhood neurological disorders require the perspective of developmental origins of disease (131). Current efforts correctly advocate for early childhood development strategies for the world’s children with disabilities (132). Greater attention to the continuities of risk affecting women and their children will help maximize benefits for all persons throughout adulthood (1).

Successful implementation of this blueprint ultimately requires global peace and prosperity. Unfortunately, there remain challenges based on persistent worldwide bio-social adversities. Diminution in poverty and related deprivations are among multiple essential goals that are required to improve brain health through education and cooperation among all stakeholders. Bio-social interventions can help reduce inequality, spur economic growth, and reduce adverse effects from climate change. Recent polycrisis events such as the Covid pandemic and regional armed conflicts reaffirm the ongoing urgency to promote universal healthcare policies to preserve brain health.

**Figure 2:**
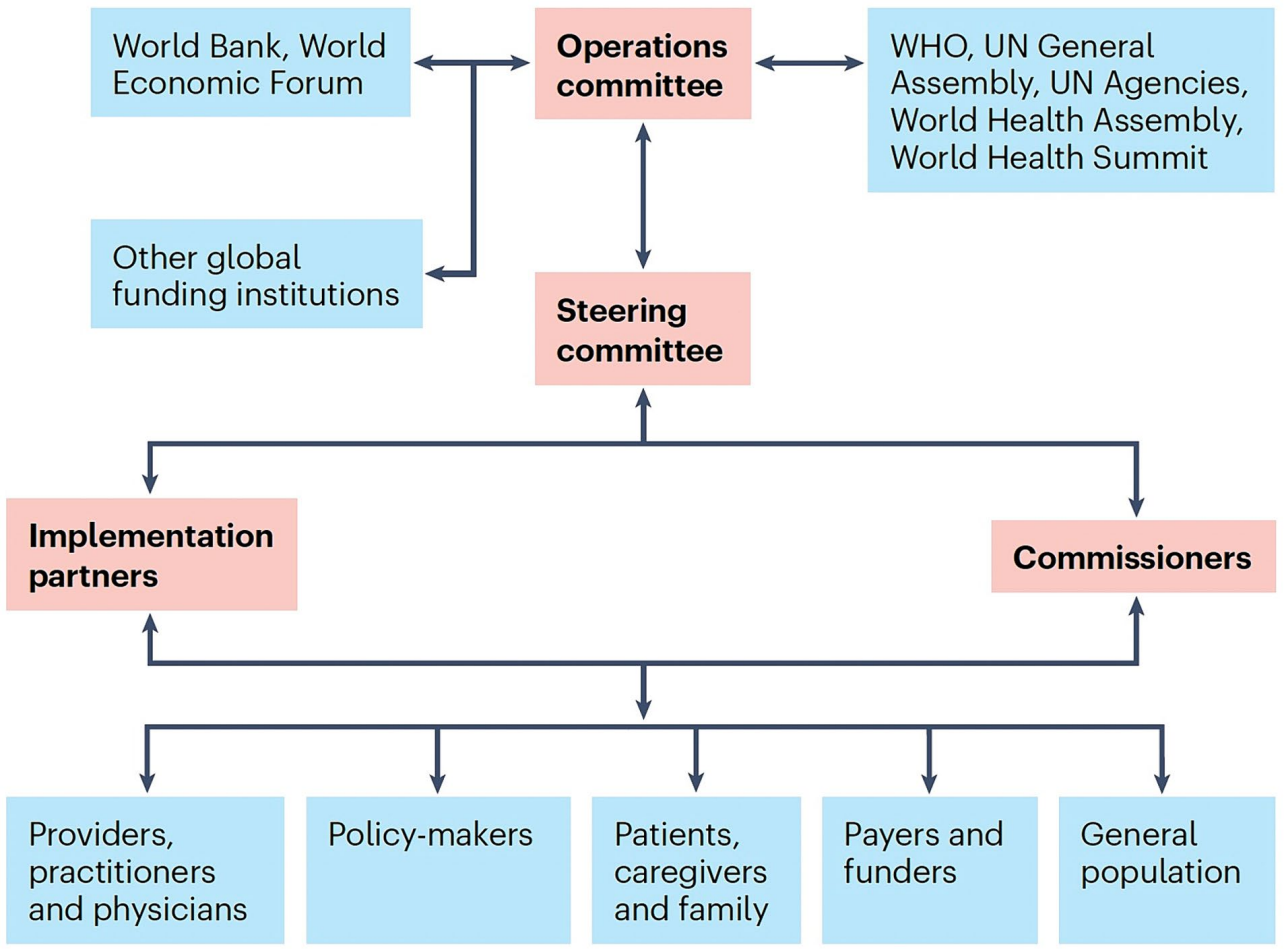
A global ecosystem is depicted that can better monitor and reduce the burden of neurological disorders with appropriate interventions. Synergistic actions among a coalition of multisector stakeholders are required to effectively address the challenge to brain health. Key stakeholders include patients, health-care service and product providers, policymakers, payers, implementation partners and the general population. Implementation partners require involvement from neurology organizations and professional societies, non-governmental agencies and ministries of health, and country ambassadors who will act as commissioners. The World Bank as well as independent funders and informed philanthropists collectively can support professional advice and funding that will optimally implement healthcare interventions. Adaptations of the global ecosystem must adjust to regional and national ecosystems to meet the demands of the respective implementation environments (13).

**Figure 3:**
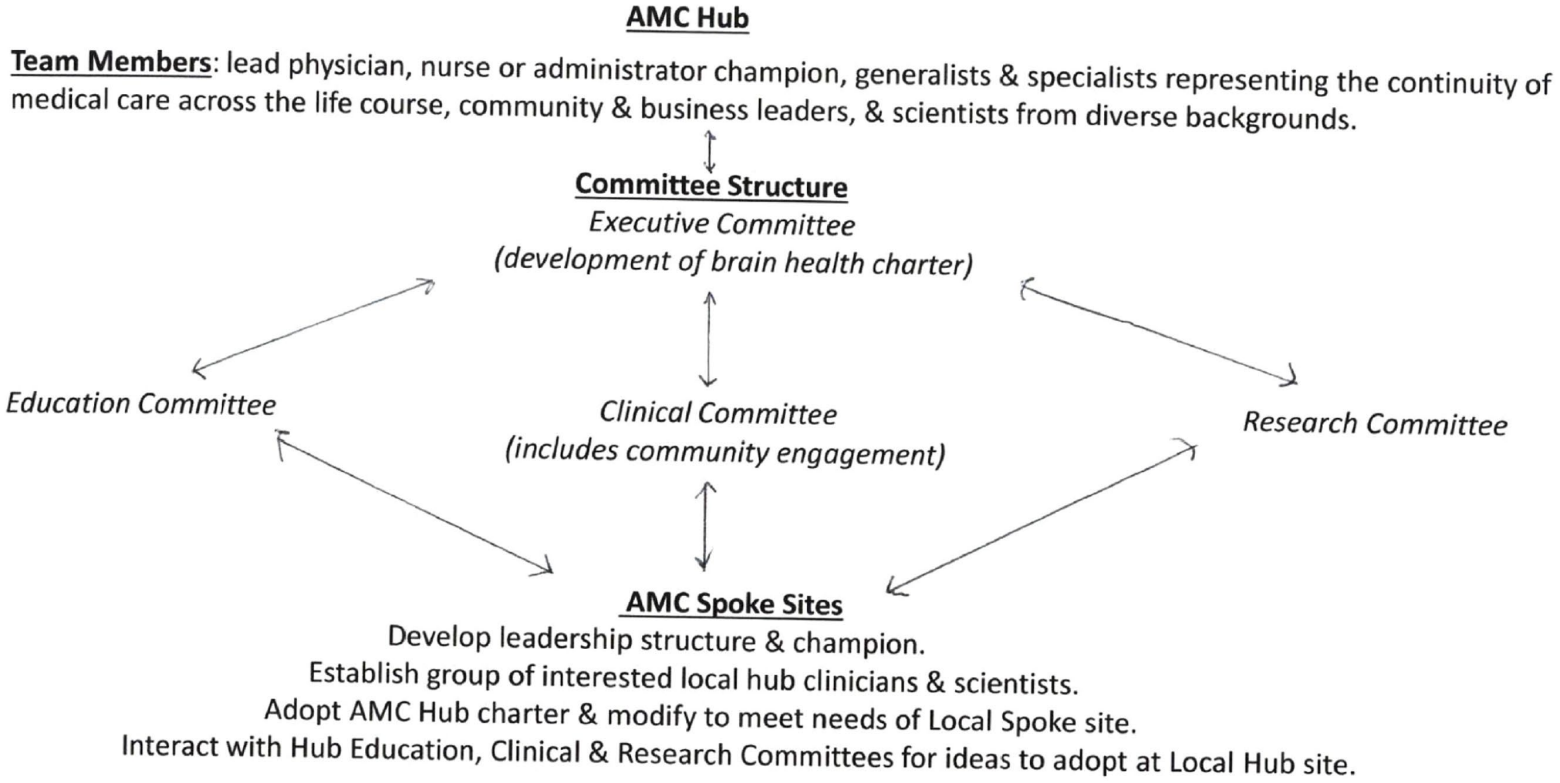
An academic center model for brain health has been proposed that creates a committee hub structure which integrates education, clinical practice, and research committee efforts. This approach supports the spokes of this hub to offer effective community, regional, national, and international collaborations. FNN training contributes to these efforts through science-based clinical and didactic activities that promote career-long practice with positive reproductive and pregnancy exposome effects on a dynamic neural exposome (14).

Global synergistic actions are proposed that will implement four key components to address healthcare: surveillance, prevention, acute interventions, and rehabilitation (13). All stakeholders through sustained cooperation can more effectively apply these actions to reduce the global burden of neurological disease. These efforts can more effectively offer positive neurologic outcomes through creativity, attention, openness, and entrepreneurship. These themes have been emphasized at a science summit sponsored by the United Nations General Assembly (15). Adjustments to address resource assets or deficiencies are specific to a community, region, or nation (Figure 2). This review of a proposed FNN training program curriculum offers an educational approach requiring experienced faculty, educational resources and motivated trainees. An academic medical center hub model can promote advances in brain health through educational leadership provided to smaller medical facilities (Figure 3) (14). A committee structure is proposed that will comprehensively address integrated clinical practice, educational and research objectives across disciplines.

Priorities to achieve these objectives must initially address vulnerable women and children who have been exposed to disease and adversity. Neurologic sequelae expressed over the lifespan consequently represent the neurological burden from diseases that will be confronted by older children and adult populations based on early disease or adversity. Interdisciplinary healthcare synergy re-enforced by this proposed FNN training program will apply the concept of a dynamic neural exposome across the lifespan to preserve or restore brain health. This science-based agenda will help mitigate or avoid TSI effects and reduce mortality and lessen neurologic morbidities into old age and across generations.

## Author contributions

MS: Conceptualization, Writing – original draft.

## Footnotes

^1^https://www.ucns.org

## References

[1] World Health Organization. Optimizing brain health across the life course; a position paper. Geneva: World Health Organization; (2022). Licence: CC BY-NC-SA 3.0 IGO; Available at: https://creativecommons.org/licenses/by-nc-sa/3.0/igo/.;2022.

[2] Rost NS Salinas J Jordan JT Banwell B Correa DJ Said RR. The brain health imperative in the 21st century—A call to action: the AAN brain health platform and position statement. Neurology. (2023) 101:570–9. doi: 10.1212/WNL.0000000000207739, PMID: 37730439 PMC10558159

[3] Scher MS. Interdisciplinary fetal-neonatal neurology training applies neural Exposome perspectives to neurology principles and practice. Front Neurol. (2023) 14:674. doi: 10.3389/fneur.2023.1321674 PMC10824035 38288328

[4] Wild CP. Complementing the genome with an “exposome”: the outstanding challenge of environmental exposure measurement in molecular epidemiology. Cancer Epidemiol Biomarkers Prev. (2005) 14:1847–50. doi: 10.1158/1055-9965.EPI-05-0456, PMID: 16103423

[5] Tamiz AP Koroshetz WJ Dhruv NT Jett DA. A focus on the neural exposome. Neuron. (2022) 110:1286–9. doi: 10.1016/j.neuron.2022.03.019, PMID: 35349785

[6] Danziger P Laventhal N. Prenatal consultation: perspectives on training, relevance, and utilization among pediatric subspecialty program directors. J Perinatol. (2018) 38:989–96. doi: 10.1038/s41372-018-0121-z, PMID: 29740188

[7] Tarui T Venkatesan C Gano D Lemmon ME Mulkey SB Pardo AC. Fetal neurology practice survey: current practice and the future directions. Pediatr Neurol. (2023) 145:74–9. doi: 10.1016/j.pediatrneurol.2023.04.016, PMID: 37290231

[8] Bjorklund DF. Ontogenetic adaptations In: Encyclopedia of evolutionary psychological science: Springer International Publishing (2016). 1–3.

[9] Stiles J Jernigan TL. The basics of brain development. Neuropsychol Rev. (2010) 20:327–48. doi: 10.1007/s11065-010-9148-4, PMID: 21042938 PMC2989000

[10] Gilmore JH Knickmeyer RC Gao W. Imaging structural and functional brain development in early childhood. Nat Rev Neurosci. (2018) 19:123–37. doi: 10.1038/nrn.2018.1, PMID: 29449712 PMC5987539

[11] Zhang P Carlsten C Chaleckis R Hanhineva K Huang M Isobe T. Defining the scope of Exposome studies and research needs from a multidisciplinary perspective. Environ Sci Technol Lett. (2021) 8:839–52. doi: 10.1021/acs.estlett.1c00648, PMID: 34660833 PMC8515788

[12] Harville EW Kruse AN Zhao Q. The impact of early-life exposures on Women’s reproductive health in adulthood. Curr Epidemiol Rep. (2021) 8:175–89. doi: 10.1007/s40471-021-00279-0, PMID: 34664023 PMC8516091

[13] Owolabi MO Leonardi M Bassetti C Jaarsma J Hawrot T Makanjuola AI. Global synergistic actions to improve brain health for human development. Nat Rev Neurol. (2023) 19:371–83. doi: 10.1038/s41582-023-00808-z, PMID: 37208496 PMC10197060

[14] Gorelick PB Hainsworth AH Wallin A. What will it take to achieve brain health globally? Cereb Circ Cogn Behav. (2024) 6:100209. doi: 10.1016/j.cccb.2024.100209, PMID: 38328025 PMC10847852

[15] European Brain Council. BRAIN CAPITAL BUILDING @ UNGA78 brain deals to harness AI and drive sustainable development goal implementation by 2030. (2023).

[16] Eyre HA Hynes W Ayadi R Manes F Swieboda P. Brain capital is crucial for global sustainable development. Lancet Neurol. (2024) 23:233–5. doi: 10.1016/S1474-4422(24)00031-0, PMID: 38280391

[17] Smith E Ali D Wilkerson B Dawson WD Sobowale K Reynolds C III. A brain capital grand strategy: toward economic reimagination. Mol Psychiatry. (2021) 26:3–22. doi: 10.1038/s41380-020-00918-w, PMID: 33100330 PMC8244537

[18] Scher MS. “The first thousand days”define a fetal/neonatal neurology program. Front Pediatr. (2021) 9:1–28. doi: 10.3389/fped.2021.683138 PMC8365757 34408995

[19] Molloy EJ El-Dib M Juul SE Benders M Gonzalez F Bearer C. Neuroprotective therapies in the NICU in term infants: present and future. Pediatr Res. (2022) 93:1819–27. doi: 10.1038/s41390-022-02295-2, PMID: 36195634 PMC10070589

[20] Sandoval Karamian AG Mercimek-Andrews S Mohammad K Molloy EJ Chang T Chau V. Neonatal encephalopathy: etiologies other than hypoxic-ischemic encephalopathy. Semin Fetal Neonatal Med. (2021) 26:101272. doi: 10.1016/j.siny.2021.101272 34417137

[21] Beck J Loron G Ancel PY Alison M Hertz Pannier L Vo van P. An updated overview of MRI injuries in neonatal encephalopathy: LyTONEPAL cohort. Children. (2022) 9:561. doi: 10.3390/children9040561, PMID: 35455605 PMC9032533

[22] Khong TY Mooney EE Ariel I Balmus NCM Boyd TK Brundler MA. Sampling and definitions of placental lesions: Amsterdam placental workshop group consensus statement. Arch Pathol Lab Med. (2016) 140:698–713. doi: 10.5858/arpa.2015-0225-CC, PMID: 27223167

[23] Lemmon ME Barks MC Bansal S Bernstein S Kaye EC Glass HC. The ALIGN framework: A parent-informed approach to prognostic communication for infants with neurologic conditions. Neurology. (2023) 100:E800–7. doi: 10.1212/WNL.0000000000201600, PMID: 36456199 PMC9984217

[24] Scher MS. The science of uncertainty guides fetal-neonatal neurology principles and practice: diagnostic-prognostic opportunities and challenges. Front Neurol. (2024) 15:933. doi: 10.3389/fneur.2024.1335933, PMID: 38352135 PMC10861710

[25] Sameroff A. A unified theory of development: A dialectic integration of nature and nurture. Child Dev. (2010) 81:6–22. doi: 10.1111/j.1467-8624.2009.01378.x, PMID: 20331651

[26] Wagen AZ Coath W Keshavan A James SN Parker TD Lane CA. Life course, genetic, and neuropathological associations with brain age in the 1946 British birth cohort: a population-based study. Lancet Healthy Longev. (2022) 3:e607–16. doi: 10.1016/S2666-7568(22)00167-2, PMID: 36102775 PMC10499760

[27] Klebanoff MA. The collaborative perinatal project: a 50-year retrospective. Paediatr Perinat Epidemiol. (2009) 23:2–8. doi: 10.1111/j.1365-3016.2008.00984.x, PMID: 19228308 PMC2646177

[28] Leibovitz Z Lerman-Sagie T Haddad L. Fetal brain development: regulating processes and related malformations. Lifestyles. (2022) 12:809. doi: 10.3390/life12060809, PMID: 35743840 PMC9224903

[29] Cater SW Boyd BK Ghate SV. Abnormalities of the fetal central nervous system: prenatal us diagnosis with postnatal correlation. Radiographics. (2020) 40:1458–72. doi: 10.1148/rg.2020200034, PMID: 32706613

[30] Pinto NM Morris SA Moon-Grady AJ Donofrio MT. Prenatal cardiac care: goals, priorities & gaps in knowledge in fetal cardiovascular disease: perspectives of the fetal heart society. Prog. Pediatr Cardiol. (2020) 59:1312. doi: 10.1016/j.ppedcard.2020.101312, PMID: 33100800 PMC7568498

[31] Lebit FD DrRadu P Florentina DL. The role of 4D ultrasound in the assessment of fetal. Behaviour. (2011) 6:120–7. PMC3239390 22205894

[32] Semeia L Sippel K Moser J Preissl H. Evaluation of parameters for fetal behavioural state classification. Sci Rep. (2022) 12:3410. doi: 10.1038/s41598-022-07476-x, PMID: 35233073 PMC8888564

[33] Carlson LM Vora NL. Prenatal diagnosis: screening and diagnostic tools. Obstet Gynecol Clin N Am. (2017) 44:245–56. doi: 10.1016/j.ogc.2017.02.004, PMID: 28499534 PMC5548328

[34] Hughes AE Sovio U Gaccioli F Cook E Charnock-Jones DS Smith GCS. The association between first trimester AFP to PAPP-A ratio and placentally-related adverse pregnancy outcome. Placenta. (2019) 81:25–31. doi: 10.1016/j.placenta.2019.04.005, PMID: 31138428

[35] Heazell AEP Hayes DJL Whitworth M Takwoingi Y Bayliss SE Davenport C. Biochemical tests of placental function versus ultrasound assessment of fetal size for stillbirth and small-for-gestational-age infants. Cochrane Database Syst Rev. (2019) 2019:CD012245. doi: 10.1002/14651858.CD012245.pub2, PMID: 31087568 PMC6515632

[36] Mavreli D Theodora M Kolialexi A. Known biomarkers for monitoring pregnancy complications. Expert Rev Mol Diagn. (2021) 21:1115–7. doi: 10.1080/14737159.2021.1971078 34429008

[37] Han VX Patel S Jones HF Dale RC. Maternal immune activation and neuroinflammation in human neurodevelopmental disorders. Nat Rev Neurol. (2021) 17:564–79. doi: 10.1038/s41582-021-00530-8 34341569

[38] Brosens I Puttemans P Benagiano G. Placental bed research: I. The placental bed: from spiral arteries remodeling to the great obstetrical syndromes. Am J Obstet Gynecol. (2019) 221:437–56. doi: 10.1016/j.ajog.2019.05.044, PMID: 31163132

[39] Para R Romero R Miller D Galaz J Done B Peyvandipour A. The distinct immune nature of the fetal inflammatory response syndrome type I and type II. Immunohorizons. (2021) 5:735–51. doi: 10.4049/immunohorizons.2100047, PMID: 34521696 PMC9394103

[40] Freedman AA Miller GE Ernst LM. Chronic villitis: refining the risk ratio of recurrence using a large placental pathology sample. Placenta. (2021) 112:135–40. doi: 10.1016/j.placenta.2021.07.298, PMID: 34352489 PMC8405570

[41] Romero R Whitten A Korzeniewski SJ Than NG Chaemsaithong P Miranda J. Maternal floor infarction/massive Perivillous fibrin deposition: A manifestation of maternal Antifetal rejection? Am J Reprod Immunol. (2013) 70:285–98. doi: 10.1111/aji.12143, PMID: 23905710 PMC4122226

[42] Harris LK Benagiano M D’Elios MM Brosens I Benagiano G. Placental bed research: II. Functional and immunological investigations of the placental bed. Am J Obstet Gynecol. (2019) 221:457–69. doi: 10.1016/j.ajog.2019.07.010, PMID: 31288009

[43] Scher MS. Neurologic outcome after fetal inflammatory response syndrome: trimester-specific considerations. Semin Fetal Neonatal Med. (2020) 25:101137. doi: 10.1016/j.siny.2020.101137, PMID: 33158496

[44] Menard MK Kilpatrick S Saade G Hollier LM Joseph GF Jr Barfield W. Levels of maternal care. Am J Obstet Gynecol. (2015) 212:259–71. doi: 10.1016/j.ajog.2014.12.030 25620372

[45] DeSisto CL Kroelinger CD Levecke M Akbarali S Pliska E Barfield WD. Maternal and neonatal risk-appropriate care: gaps, strategies, and areas for further research. J Perinatol. (2023) 43:817–22. doi: 10.1038/s41372-022-01580-6, PMID: 36631565 PMC9838520

[46] Nelson HD Darney BG Ahrens K Burgess A Jungbauer RM Cantor A. Associations of unintended pregnancy with maternal and infant health outcomes: A systematic review and Meta-analysis. JAMA. (2022) 328:1714–29. doi: 10.1001/jama.2022.19097, PMID: 36318133 PMC9627416

[47] Diabelková J Rimárová K Dorko E Urdzík P Houžvičková A Argalášová Ľ. Adolescent pregnancy outcomes and risk factors. Int J Environ Res Public Health. (2023) 20:113. doi: 10.3390/ijerph20054113, PMID: 36901128 PMC10002018

[48] Kissler K Hurt KJ. The pathophysiology of labor dystocia: theme with variations. Reprod Sci. (2023) 30:729–42. doi: 10.1007/s43032-022-01018-6, PMID: 35817950 PMC10388369

[49] Drennan KJ Vanushkina M. Chapter 9: principles of reproductive healthcare in chronic neurologic disease In: Ciafaloni E, editor. Neurological diseases and pregnancy: A coordinated care model for best management. 1st ed: Oxford University Press-Academic. (2018).

[50] Croskerry P. The importance of cognitive errors in diagnosis and strategies to minimize them. Acad Med. (2003) 78:775–80. doi: 10.1097/00001888-200308000-00003, PMID: 12915363

[51] Lear CA Wassink G Westgate JA Nijhuis JG Ugwumadu A Galinsky R. The peripheral chemoreflex: indefatigable guardian of fetal physiological adaptation to labour. J Physiol. (2018) 596:5611–23. doi: 10.1113/JP274937, PMID: 29604081 PMC6265558

[52] Lear CA Kasai M Booth LC Drury PP Davidson JO Maeda Y. Peripheral chemoreflex control of fetal heart rate decelerations overwhelms the baroreflex during brief umbilical cord occlusions in fetal sheep. J Physiol. (2020) 598:4523–36. doi: 10.1113/JP279573, PMID: 32705685

[53] Vrabie SC Novac L Manolea MM Dijmarescu LA Novac M Siminel MA. Abnormalities of the umbilical cord In: Congenital anomalies-from the embryo to the neonate. Stefania T. InTech (2018).

[54] Rees C van Vliet H Siebers A Bulten J Huppelschoten A Westerhuis M. The ADENO study: ADenomyosis and its effect on neonatal and obstetric outcomes: a retrospective population-based study. Am J Obstet Gynecol. (2022) 229:49.e1–49.e12. doi: 10.1016/j.ajog.2022.12.013, PMID: 36539028

[55] Anderson R Bin ZS Limmer M. The impact of introducing midwives and also mentoring on the quality of sexual, reproductive, maternal, newborn, and adolescent health Services in low-and Middle-Income Countries: An Integrative Review. Protocol Methods Protoc. (2023) 6:48. doi: 10.3390/mps6030048, PMID: 37218908 PMC10204447

[56] Hill MG Reed KL Brown RN. Perinatal asphyxia from the obstetric standpoint. Semin Fetal Neonatal Med. (2021) 26:101259. doi: 10.1016/j.siny.2021.101259 34175240

[57] Sartwelle TP Johnston JC Arda B Zebenigus M. Electronic fetal monitoring in the twenty-first century: language, logic and Lewis Carroll. Clin Ethics. (2021) 16:213–21. doi: 10.1177/1477750920971800

[58] Jacobs SE Berg M Hunt R Tarnow-Mordi WO Inder TE Davis PG. Cooling for newborns with hypoxic ischaemic encephalopathy. Cochrane Database Syst Rev. (2013) 2013:CD003311. doi: 10.1002/14651858.CD003311.pub3, PMID: 23440789 PMC7003568

[59] Mathew JL Kaur N Dsouza JM. Therapeutic hypothermia in neonatal hypoxic encephalopathy: A systematic review and meta-analysis. J Glob Health. (2022) 12:4030. doi: 10.7189/JOGH.12.04030, PMID: 35444799 PMC8994481

[60] Sarnat HB Flores-Sarnat L Fajardo C Leijser LM Wusthoff C Mohammad K. Grading scale for neonatal encephalopathy (Sarnat & Sarnat 1976): 45 year update proposal. Pediatr Neurol. (2020) 113:75–9. doi: 10.1016/j.pediatrneurol.2020.08.014, PMID: 33069006

[61] Nakwa FL Sepeng L van Kwawegen A Thomas R Seake K Mogajane T. Characteristics and outcomes of neonates with intrapartum asphyxia managed with therapeutic hypothermia in a public tertiary hospital in South Africa. BMC Pediatr. (2023) 23:51. doi: 10.1186/s12887-023-03852-2, PMID: 36721127 PMC9890846

[62] Wu YW Comstock BA Gonzalez FF Mayock DE Goodman AM Maitre NL. Trial of erythropoietin for hypoxic-ischemic encephalopathy in newborns. N Engl J Med. (2022) 387:148–59. doi: 10.1056/nejmoa2119660, PMID: 35830641 PMC10542745

[63] Wu YW Goodman AM Chang T Mulkey SB Gonzalez FF Mayock DE. Placental pathology and neonatal brain MRI in a randomized trial of erythropoietin for hypoxic–ischemic encephalopathy. Pediatr Res. (2020) 87:879–84. doi: 10.1038/s41390-019-0493-6, PMID: 31261373

[64] Scher MS. Neonatal encephalopathy is a complex phenotype representing reproductive and pregnancy Exposome effects on the maternal-placental-fetal triad. Clin Perinatol. (2024). doi: 10.1016/j.clp.2024.04.001 39095094

[65] Ginsberg Y Khatib N Weiner Z Beloosesky R. Maternal inflammation, fetal brain implications and suggested neuroprotection: A summary of 10 years of research in animal models. Rambam Maimonides Med J. (2017) 8:e0028. doi: 10.5041/rmmj.10305, PMID: 28467756 PMC5415374

[66] Kelly SB Tran NT Polglase GR Hunt RW Nold MF Nold-Petry CA. A systematic review of immune-based interventions for perinatal neuroprotection: closing the gap between animal studies and human trials. J Neuroinflammation. (2023) 20:241. doi: 10.1186/s12974-023-02911-w, PMID: 37864272 PMC10588248

[67] Redline RW. Placental pathology: pathways leading to or associated with perinatal brain injury in experimental neurology, special issue: placental mediated mechanisms of perinatal brain injury. Exp Neurol. (2022) 347:113917. doi: 10.1016/j.expneurol.2021.113917 34748755

[68] Redline RW Roberts DJ Parast MM Ernst LM Morgan TK Greene MF. Placental pathology is necessary to understand common pregnancy complications and achieve an improved taxonomy of obstetrical disease. Am J Obstet Gynecol. (2023) 228:187–202. doi: 10.1016/j.ajog.2022.08.010, PMID: 35973475 PMC10337668

[69] Chan JSY Baergen RN. Gross umbilical cord complications are associated with placental lesions of circulatory stasis and fetal hypoxia. Pediatr Dev Pathol. (2012) 15:487–94. doi: 10.2350/12-06-1211-OA.1, PMID: 22978619

[70] Parmentier CEJ de Vries LS Groenendaal F. Magnetic resonance imaging in (near-)term infants with hypoxic-ischemic encephalopathy. Diagnostics. (2022) 12:645. doi: 10.3390/diagnostics12030645, PMID: 35328199 PMC8947468

[71] Levison SW Rocha-Ferreira E Kim BH Hagberg H Fleiss B Gressens P. Mechanisms of tertiary neurodegeneration after neonatal hypoxic-ischemic brain damage. Pediatr Med. (2022) 5:28. doi: 10.21037/pm-20-104, PMID: 37601279 PMC10438849

[72] Goldberg H. Unraveling neurodiversity: insights from neuroscientific perspectives. Encyclopedia. (2023) 3:972–80. doi: 10.3390/encyclopedia3030070

[73] Cardona VQ Cohen S Cook N Cizmeci MN Chandel A DiGeronimo R. The current state of neonatal neurodevelopmental follow-up programs in North America: A Children’s hospitals neonatal consortium report. Am J Perinatol. (2024). doi: 10.1055/a-2283-8843, PMID: 38458236

[74] Bhutta ZA Bhavnani S Betancourt TS Tomlinson M Patel V. Adverse childhood experiences and lifelong health. Nat Med. (2023) 29:1639–48. doi: 10.1038/s41591-023-02426-0, PMID: 37464047

[75] Fitzallen GC Taylor HG Bora S. What do we know about the preterm behavioral phenotype? A narrative review. Front Psych. (2020) 11:154. doi: 10.3389/fpsyt.2020.00154, PMID: 32269532 PMC7109291

[76] Pellmar TC Eisenberg L. Bridging disciplines in the brain, behavioral, and clinical sciences National Academies Press, US (2000). 20669407

[77] Leonardi M Lee H Kostanjsek N Fornari A Raggi A Martinuzzi A. 20 years of ICF—international classification of functioning, disability and health: uses and applications around the world. Int J Environ Res Public Health. (2022) 19:321. doi: 10.3390/ijerph191811321, PMID: 36141593 PMC9517056

[78] Ustin TNB. WHO’s ICF and functional status information in health records. Healthc Fin Rev. (2003) 24:77–88. PMC4194828 12894636

[79] Kostanjsek N. Use of the international classification of functioning, disability and health (ICF) as a conceptual framework and common language for disability statistics and health information systems. BMC Public Health. (2011) 11:S3. doi: 10.1186/1471-2458-11-S4-S3 PMC3104216 21624189

[80] National Academies of Sciences, Engineering, and Medicine; Division of Behavioral and Social Sciences and Education; Health and Medicine Division; Committee on Population; Board on Health Sciences Policy; Committee on the Use of Race, Ethnicity, and Ancestry as Population Descriptors in Genomics Research. Using population descriptors in genetics and genomics research: A new framework for an evolving field. National Academies Press, US (2023). 36989389

[81] Matta C. Neuroscience and educational practice–A critical assessment from the perspective of philosophy of science. Educ Philos Theory. (2021) 53:197–211. doi: 10.1080/00131857.2020.1773801

[82] Dresler T Bugden S Gouet C Lallier M Oliveira DG Pinheiro-Chagas P. A translational framework of educational neuroscience in learning disorders. Front Integr Neurosci. (2018) 12:25. doi: 10.3389/fnint.2018.00025, PMID: 30022931 PMC6039789

[83] Wilcox G Morett LM Hawes Z Dommett EJ. Why educational neuroscience needs educational and school psychology to effectively translate neuroscience to educational practice. Front Psychol. (2021) 11:449. doi: 10.3389/fpsyg.2020.618449, PMID: 33519642 PMC7840578

[84] Thomas MSC Ansari D Knowland VCP. Annual research review: educational neuroscience: progress and prospects. J Child Psychol Psychiatry. (2019) 60:477–92. doi: 10.1111/jcpp.12973, PMID: 30345518 PMC6487963

[85] National Academies. Transforming the workforce for children birth through age 8 National Academies Press (2015). 26269871

[86] Brown TT Jernigan TL. Brain development during the preschool years. Neuropsychol Rev. (2012) 22:313–33. doi: 10.1007/s11065-012-9214-1, PMID: 23007644 PMC3511633

[87] Department of Education. Assistance to states for the education of children with disabilities. Fed Regist. (2023) 88:31659–67.

[88] National Coordinating Center for Communications, US Department of Homeland Security, Cybersecurity and Intrastructure Security Agency. (2020).

[89] Inder TE Volpe JJ Anderson PJ. Defining the neurologic consequences of preterm birth. N Engl J Med. (2023) 389:441–53. doi: 10.1056/nejmra2303347, PMID: 37530825

[90] Robinson R Lahti-Pulkkinen M Schnitzlein D Voit F Girchenko P Wolke D. Mental health outcomes of adults born very preterm or with very low birth weight: A systematic review. Semin Fetal Neonatal Med. (2020) 25:101113. doi: 10.1016/j.siny.2020.101113 32402835

[91] Smythe T Kuper H. The association between disability and all-cause mortality in low-income and middle-income countries: a systematic review and meta-analysis. Lancet Glob Health. (2024) 12:e756–70. doi: 10.1016/S2214-109X(24)00042-1, PMID: 38614629

[92] Scher MS. A bio-social model during the first 1000 days optimizes healthcare for children with developmental disabilities. Biomedicines. (2022) 10:290. doi: 10.3390/biomedicines10123290, PMID: 36552046 PMC9775202

[93] Institute of Education for Healthcare and Medical Sciences. Standard Operating Procedure/Guideline. (2022).

[94] Mubuuke AG Mwesigwa C Kiguli S. Implementing the Angoff method of standard setting using postgraduate students: practical and affordable in resource-limited settings. Afr J Health Prof Educ. (2017) 9:171–5. doi: 10.7196/ajhpe.2017.v9i4.631, PMID: 29291132 PMC5745345

[95] Gelb DJ Kraakevik J Safdieh JE Agarwal S Odia Y Govindarajan R. Contemporary neuroscience Core curriculum for medical schools. Neurology. (2021) 97:675–84. doi: 10.1212/WNL.0000000000012664, PMID: 34400582 PMC8520386

[96] Young M St-Onge C Xiao J Vachon Lachiver E Torabi N. Characterizing the literature on validity and assessment in medical education: a bibliometric study. Perspect Med Educ. (2018) 7:182–91. doi: 10.1007/s40037-018-0433-x, PMID: 29796976 PMC6002290

[97] Helou MA Diaz Granados D Ryan MS Cyrus JW. Uncertainty in decision making in medicine: A scoping review and thematic analysis of conceptual models. Acad Med. (2020) 95:157–65. doi: 10.1097/ACM.0000000000002902, PMID: 31348062 PMC6925325

[98] Hguyen A Van Meijgaard J Kim S Marsh T. Mapping healthcare deserts. The GoodRx Research Team, Lipincott (2021).

[99] Brigance C. Lucas R. Jones E. Davis A. Oinuma M. Mishkin K.. Nowhere to go: Maternity care deserts across the U.S. (report no. 3). March of Dimes; (2022).

[100] Oh SS Galanter J Thakur N Pino-Yanes M Barcelo NE White MJ. Diversity in clinical and biomedical research: A promise yet to be fulfilled. PLoS Med. (2015) 12:e1001918. doi: 10.1371/journal.pmed.1001918, PMID: 26671224 PMC4679830

[101] Boyce WT Sokolowski MB Robinson GE. Genes and environments, development and time. Proc Natl Acad Sci USA. (2020) 117:23235–41. doi: 10.1073/pnas.2016710117, PMID: 32967067 PMC7519332

[102] Bain L Norris SP Stroud C. Environmental Neuroscience National Academies Press (2020). 33180402

[103] Vineis P Barouki R. The exposome as the science of social-to-biological transitions. Environ Int. (2022) 165:107312. doi: 10.1016/j.envint.2022.107312, PMID: 35635963

[104] Jimma BL. Artificial intelligence in healthcare: A bibliometric analysis. Telemat Inform Rep. (2023) 9:100041. doi: 10.1016/j.teler.2023.100041

[105] Cachat-Rosset G Klarsfeld A. Diversity, Equity, and inclusion in artificial intelligence: an evaluation of guidelines. Appl Artif Intell. (2023) 37:6618. doi: 10.1080/08839514.2023.2176618

[106] Orchard ER Rutherford HJV Holmes AJ Jamadar SD. Matrescence: lifetime impact of motherhood on cognition and the brain. Trends Cogn Sci. (2023) 27:302–16. doi: 10.1016/j.tics.2022.12.002, PMID: 36609018 PMC9957969

[107] Scher MS. Fetal neurology: principles and practice with a life-course perspective. Handb Clin Neurol. (2019) 162:1–29. doi: 10.1016/B978-0-444-64029-1.00001-1, PMID: 31324306

[108] Köhler S Gargano M Matentzoglu N Carmody LC Lewis-Smith D Vasilevsky NA. The human phenotype ontology in 2021. Nucleic Acids Res. (2021) 49:D1207-D 1217. doi: 10.1093/nar/gkaa1043, PMID: 33264411 PMC7778952

[109] Bhattacharya A Freedman AN Avula V Harris R Liu W Pan C. Placental genomics mediates genetic associations with complex health traits and disease. Nat Commun. (2022) 13:706. doi: 10.1038/s41467-022-28365-x, PMID: 35121757 PMC8817049

[110] De Asis-Cruz J Andescavage N Limperopoulos C. Adverse prenatal exposures and fetal brain development: Insights from advanced fetal magnetic resonance imaging. Biol Psychiatry Cogn Neurosci Neuroimaging. (2021) 7:480–90. doi: 10.1016/j.bpsc.2021.11.009 34848383

[111] Thomason ME Hect JL Waller R Curtin P. Interactive relations between maternal prenatal stress, fetal brain connectivity, and gestational age at delivery. Neuropsychopharmacology. (2021) 46:1839–47. doi: 10.1038/s41386-021-01066-7, PMID: 34188185 PMC8357800

[112] Chakraborty S Parayil R Mishra S Nongthomba U Clement JP. Epilepsy characteristics in neurodevelopmental disorders: research from patient cohorts and animal models focusing on autism Spectrum disorder. Int J Mol Sci. (2022) 23:807. doi: 10.3390/ijms231810807, PMID: 36142719 PMC9501968

[113] Monk C Lugo-Candelas C Trumpff C. Prenatal developmental origins of future psychopathology: mechanisms and pathways. Annu Rev Clin Psychol. (2019) 15:317–44. doi: 10.1146/annurev-clinpsy-050718-095539, PMID: 30795695 PMC7027196

[114] Tartaglione AM Venerosi A Calamandrei G. Early-life toxic insults and onset of sporadic neurodegenerative diseases—an overview of experimental studies. Curr Top Behav Neurosci. (2016) 29:231–64. doi: 10.1007/7854_2015_416, PMID: 26695168

[115] de Rooij SR. Are brain and cognitive reserve shaped by early life circumstances? Front Neurosci. (2022) 16:5811. doi: 10.3389/fnins.2022.825811, PMID: 35784851 PMC9243389

[116] Oliveira MAP Balling R Smidt MP Fleming RMT. Embryonic development of selectively vulnerable neurons in Parkinson’s disease. NPJ Parkinsons Dis. (2017) 3:21. doi: 10.1038/S41531-017-0022-4, PMID: 28685157 PMC5484687

[117] Jiménez-Salvador I Meade P Iglesias E Bayona-Bafaluy P Ruiz-Pesini E. Developmental origins of Parkinson disease: improving the rodent models. Ageing Res Rev. (2023) 86:101880. doi: 10.1016/j.arr.2023.101880, PMID: 36773760

[118] Birbeck GL Meyer AC Ogunniyi A. Nervous system disorders across the life course in resource-limited settings. Nature. (2015) 527:S167–71. doi: 10.1038/nature16031, PMID: 26580323 PMC5845480

[119] Calabrese M Magliozzi R Ciccarelli O Geurts JJG Reynolds R Martin R. Exploring the origins of grey matter damage in multiple sclerosis. Nat Rev Neurosci. (2015) 16:147–58. doi: 10.1038/nrn3900, PMID: 25697158

[120] Prüss H. Autoantibodies in neurological disease. Nat Rev Immunol. (2021) 21:798–813. doi: 10.1038/s41577-021-00543-w, PMID: 33976421 PMC8111372

[121] Muller M Sigurdsson S Kjartansson O Gunnarsdottir I Thorsdottir I Harris TB. Late-life brain volume: A life-course approach. The AGES-Reykjavik study. Neurobiol Aging. (2016) 41:86–92. doi: 10.1016/j.neurobiolaging.2016.02.012, PMID: 27103521 PMC5751431

[122] Elliott RO He M. Unlocking the power of exosomes for crossing biological barriers in drug delivery. Pharmaceutics. (2021) 13:1–20. doi: 10.3390/pharmaceutics13010122, PMID: 33477972 PMC7835896

[123] Bai K Li X Zhong J Ng EHY Yeung WSB Lee CL. Placenta-derived exosomes as a modulator in maternal immune tolerance during pregnancy. Front Immunol. (2021) 12:1093. doi: 10.3389/fimmu.2021.671093, PMID: 34046039 PMC8144714

[124] Abdelsalam M Ahmed M Osaid Z Hamoudi R Harati R. Insights into exosome transport through the blood-brain barrier and the potential Therapeutical applications in brain diseases. Pharmaceuticals. (2023) 16:571. doi: 10.3390/ph16040571, PMID: 37111328 PMC10144189

[125] Hu H Iyer HS Zheng Y Abdelnaser A. Climate change, human health, and the Exposome: Utilizing OMIC technologies to navigate an era of uncertainty. Front Public Health. (2022) 10:973000. doi: 10.3389/fpubh.2022.973000 36211706 PMC9533016

[126] Lu YC Kapse K Andersen N Quistorff J Lopez C Fry A. Association between socioeconomic status and in utero fetal brain development. JAMA Netw Open. (2021) 4:e213526. doi: 10.1001/jamanetworkopen.2021.3526, PMID: 33779746 PMC8008281

[127] Ramphal B Whalen DJ Kenley JK Yu Q Smyser CD Rogers CE. Brain connectivity and socioeconomic status at birth and externalizing symptoms at age 2 years. Dev Cogn Neurosci. (2020) 45:100811. doi: 10.1016/j.dcn.2020.100811, PMID: 32823180 PMC7451824

[128] Gellci K Marusak HA Peters C Elrahal F Iadipaolo AS Rabinak CA. Community and household-level socioeconomic disadvantage and functional organization of the salience and emotion network in children and adolescents. NeuroImage. (2019) 184:729–40. doi: 10.1016/j.neuroimage.2018.09.077, PMID: 30287301 PMC6230495

[129] United Nations. The-Sustainable-Development-Goals-Report-2023. (2023).

[130] Steinmetz JD Seeher KM Schiess N Nichols E Cao B Servili C. Global, regional, and national burden of disorders affecting the nervous system, 1990–2021: a systematic analysis for the global burden of disease study 2021. Lancet Neurol. (2024) 23:344–81. doi: 10.1016/S1474-4422(24)00038-3, PMID: 38493795 PMC10949203

[131] Newton CR. Global burden of pediatric neurological disorders. Semin Pediatr Neurol. (2018) 27:10–5. doi: 10.1016/j.spen.2018.03.002, PMID: 30293585 PMC7613506

[132] Olusanya BO Wright SM Smythe T Khetani MA Moreno-Angarita M Gulati S. Early childhood development strategy for the world’s children with disabilities. Front. Public Health. (2024) 12:107. doi: 10.3389/fpubh.2024.1390107 PMC11220280 38962774

