# Supplementary material for: Interdisciplinary fetal-neonatal neurology training improves brain health across the lifespan
Source: Front Neurol. 2024 Jul 4;15:1411987. doi: 10.3389/fneur.2024.1411987 (PMC11254674; doi:10.3389/fneur.2024.1411987)
Supplement: Supplementary file 1 [file Data_Sheet_1.PDF]

APPENDIX 1A –CURRICULUM TOPICS FOR "BEDSIDE" AND "DIDACTIC" NEONATAL NEUROCRITICAL CARE TRAINING APPLIED TO FETAL-NEONATAL NEUROLOGY *

CONCEPT OVERVIEW
1. Neural exposome-endogenous/exogenous stressor interplay
2. Gene-environment interactions
3. Developmental neuroplasticity
4. Neuroepidemiology (e.g. developmental disorders)
5. Developmental origins of health- disease
6. Interdisciplinary collaboration
7. Life-course theory
8. Social determinants of health
9. Bioethical principles and practice
10. Science of uncertainty
11. Cognitive-based decision making
12. Sources of bias-debiasing strategies

PERIPARTUM/LABOR AND DELIVERY
1. Maternal reportage
2. Antepartum/intrapartum fetal surveillance
3. Resuscitation strategies and test choices
4. Stabilization and initiation of interventions

SERIAL EXAMINATIONS-ASSESSMENTS
1. Apgar, Sarnat, Thompson scoring techniques
2. Anthropometric measure comparisons
3. Vital signs-autonomic nervous system assessment
4. System-specific assessments
5. Specific neurologic exam components
6. Sign and symptom evolution over time
7. System-specific testing choices
8. Pain and stress assessments
9. Developmental care assessments
10. Discharge planning coordinated with primary care and early intervention programs.

MULTI-SYSTEMIC DISORDERS (PARTIAL LIST)
1. Complex congenital heart disease
2. Pulmonary hypertension
3. Bronchopulmonary dysplasia followed by chronic lung disease
4. Sepsis
5. Fetal inflammatory response syndrome
6. System specific anomalies
7. Gastrointestinal disorders (e.g. necrotizing enterocolitis
8. Hydrops fetalis-immune/non-immune types
9. Hepatobiliary diseases
10. Metabolic/toxic disturbances
11. Neonatal abstinence syndrome
12. Fetal-maternal hemorrhage
13. Maternal medication/stressor effects

NEURODIAGNOSTIC TOOLS
1. Neuroimaging-cranial sonography, brain CT and MRI studies, MRS, computer imaging choices
2. Neurophysiological studies- continuous (video) EEG, aEEG, EEG trend algorithms, evoked response studies
3. Near-infrared spectroscopy
4. Future diagnostic choices (e.g. optical imaging)
5. Placental-cord-uterine pathology- gross/microscopic
6. Neuropathological correlates of disease
7. Multisystem histopathology and autopsy findings

NEONATAL ENCEPHALOPATHY (NE)
1. Antepartum, peripartum, intrapartum timing
2. Divers etiopathogenetic pathways
3. NE mimicry
4. Encephalopathy of prematurity

NEONATAL SEIZURES
1. Multi-tiered classification; recognition, localization, timing, etiopathogenesis
2. Neuromonitoring-conventional, continuous, amplitude integrated techniques
3. Paroxysmal (non-epileptic) disorders
4. Treatment/discontinuation decisions using antiepileptic medications.
5. Risks for epilepsy and co-morbidities
6. Genetic biomarkers for epilepsy risk

CEREBROVASCULAR DISORDERS
1. Incidence-prevalence
2. Arterial-venous developmental origins
3. Etiopathogenetic pathways
4. Neurodiagnostic evaluations
5. Intracranial hemorrhages- full term, preterm
6. Co-morbidities and prognosis

CNS MALDEVELOPMENT
1. Cerebrum – malformations based on disorders of neurogenesis, including axonal and synaptic development
2. Midline lesions: holoprosencephaly, corpus callosum, septum pellucidum defects
3. Posterior fossa structures brainstem/cerebellar midline-hemispheric
4. Anomalous versus destructive lesions
5. Spinal cord development (e.g., neural tube defects, caudal regression)
6. Diverse presentations of ventriculomegaly

CENTRAL-PERIPHERAL INJURIES
1. Craniocerebral trauma – extradural, subdural subgaleal hemorrhages,
2. Skull fracture categories
3. Brachial plexus injuries
4. Spinal cord injuries
5. Cranial and peripheral nerve injuries

NEUROGENETICS
1. Chromosomal & post translational genetic evaluations
2. Neurocutaneous disorders
3. Inborn errors of metabolism by pathway
4. Neurodegenerative diseases
5. Post-translational mechanisms-mosaicism, somatic variation, epigenetics

NEUROMUSCULAR
1. Lower motor neuron pathway development
2. Neonatal hypotonia-hypertonia phenotypes
3. Arthrogryposis spectrum
4. Spinal muscular atrophy
5. Congenital myopathies
6. Critical illness myopathy

NEUROPROTECTION
1. Neuromonitoring devices/procedures
2. Preventive, rescue, reparative options
3. Pharmacologic interventions
4. Therapeutic hypothermia-EPO
5. Non-pharmacologic interventions
6. Developmental care choices
7. Novel treatment investigations (e.g. exosomes)

PARENTAL-FAMILY INTERACTIONS
1. Medical-based decisions
2. Value-based decisions
3. Shared decision making
4. Parent-centered Outcome Research initiatives (i.e. PCORI)
5. Bioethical standards
6. Palliative care/end of life decisions
7. Autopsy requests with family conferences.

*Modified from [3]

APPENDIX 1B –REPRODUCTIVE AND PREGNANCY HEALTH COMPONENTS OF FETAL NEUROLOGY TRAINING

PRE-CONCEPTION
1. Adolescent-related pregnancy complications
2. Hypertensive disorders
3. Metabolic/endocrine disorders
4. Obesity and related disorders (PCOS)
5. Neuropsychiatric disorders & medications
6. Epilepsy & antiepileptic medications
7. Chronic neurological disorders (e.g., multiple sclerosis, neuromuscular diseases)
8. Multisystemic genetic disorders
9. Male-specific disease conditions

FERTILITY ISSUES & TESTING
1. Pre-implantation genetic diagnosis
2. Infertility
3. Artificial reproductive technologies

PRENATAL SCREENING
1. Maternal serum screening
2. First trimester screening
3. Cell free DNA / noninvasive prenatal testing (NIPT)
4. Neonatal Autoimmune Thrombocytopenia (NAIT) testing
5. TORCH testing
6. Carrier testing
7. Thrombophilia
8. Amniocentesis/chorionic villus sampling
9. Sonographic arthrometry, Doppler, Biophysical scales
10. Fetal MRIs
11. Fetal magnetoencephalogram (MEG)

NEURAL DEVELOPMENT
1. Embryonic and fetal structures
2. Range of malformations
3. Neural tube defects
4. Ventriculomegaly
5. Omics approach

GROWTH ISSUES
1. Normative growth curves
2. Fetal growth restriction
3. Microcephaly
4. Oligo-polyhydramnios

FETAL FUNCTIONAL NEURODEVELOPMENT
1. Fetal movements
2. State development/organization
3. Autonomic functions
4. Disease correlates; fetal seizures

PERINATAL PATHOLOGY
1. Developmental anatomy- function, “omics” biomarkers
2. Gross pathology -placenta, cord, uterus
3. Histopathology: chorioamnionitis-funisitis, malperfusion, villitis, villous dysmaturity
4. Neuropathological correlates with placental pathological processes.

CHROMOSOMAL & GENETIC SYNDROMES
1. Trisomies – 21; 13; 18
2. Microarray abnormalities
3. Rapid WES/GWGS testing
4. Epigenetic advances-methylome

VASCULOGENESIS/ANGIOGENESIS
1. Arterial/venous development
2. Intraventricular hemorrhages
3. Intraparenchymal hemorrhages
4. Extracranial hemorrhages
5. Ischemic /hemorrhagic stroke syndromes
6. Vascular malformations

INFECTIONS
1. TORCH
2. HIV
3. Zika virus
4. SARS 2
5. Parvovirus
6. Lymphocytic choriomeningitis virus

NEUROMUSCULAR SYSTEM
1. Clubfoot
2. Arthrogryposis
3. Fetal dyskinesia syndrome
4. Genetic neuromuscular diseases

CONNATAL TUMORS
1. Tuberous sclerosis
2. Neurocutaneous melanosis
3. Germ cell tumors – teratoma
4. Supra-infratentorial tumors

COUNSELLING AND END OF LIFE ISSUES
1. Termination – state & federal laws
2. Still births
3. Postnatal family planning
4. Recurrence risk counseling
5. Comfort or unretaliative care
6. Autopsy requests and genetic testing

FETAL THERAPIES
1. Fetal infections for TORCH,HIV
2. Maternal treatments -autoimmune, metabolic, nutritional disorders
3. Fetal surgery – myelomeningocele; tracheoesophageal fistula; congenital diaphragmatic hernia
4. Vascular – arteriovenous malformations; lymphatic malformations
5. Novel proposed protocols.

*Modified from reference[3]

APPENDIX 1C–PEDIATRIC COMPONENT (<2 years of age)*

OUTPATIENT/INPATIENT SERVICES
1. NICU discharge planning
2. PCP referrals-wellness programs
3. Early intervention services
4. Pediatric subspecialty referrals
5. Medically fragile child
6. Adverse childhood experiences
7. Hospitalizations with complications
8. PICU admissions
9. Surgical needs

STANDARDIZED ASSESSMENT TOOLS
1. Neonatal
2. Neurobehavioral
3. Gross motor
4. Speech/Language
5. Cognitive testing
6. Social-adaptive testing

HEARING ASSESSMENTS
1. Audiologist evaluations
2. Otoacoustic emissions
3. Brainstem evoked responses

VISION ASSESSMENTS
1. Visual acuity and fields
2. Retinopathy of prematurity
4. Cerebral visual impairment

OUTCOMES ASSESSMENT
1. Vision impaired
2. Hearing impaired
3. Global developmental delay
4. Cerebral palsy
5. Neurobehavior– DSM-TR 5 criteria
6. Cognitive skill testing-executive function
7. Intellectual disability categories
8. DSM-5 classification of mental health Disorders (including TR revisions for autistic spectrum etc.)

THERAPIES & REHABILITATION
1. Physical therapy
2. Occupational therapy
3. Speech/language therapy
4. Nutrition/feeding therapy
5. Aqua therapy
6. Vision therapy
7. Computer-assisted communication

*Modified from reference [3]
