# Supplementary material for: Interdisciplinary fetal-neonatal neurology training improves brain health across the lifespan
Source: Front Neurol. 2024 Jul 4;15:1411987. doi: 10.3389/fneur.2024.1411987 (PMC11254674; doi:10.3389/fneur.2024.1411987)
Supplement: Supplementary file 2 [file Data_Sheet_2.PDF]

APPENDIX 2: FETAL-NEONATAL NEUROLOGY TOPICS FOR EXAMINATION QUESTIONS*

Curriculum Topic Headings (see Appendix 1)		Percentage of Questions
I.	Concepts and Diagnostic Approaches	5%
II	Neonatal Neurocritical Care	45%
III.	Reproductive/pregnancy care.	10%
IV.	Fetal Neurology	25%
IV.	Pediatric follow up Care	15%

I. Concepts and Diagnostic Approaches

1. Developmental origins
2. Life-course theory
3. Cognitive decision-making process
4. Neural Exposome in relation to reproductive and pregnancy exposome effects
5. Interdisciplinary collaborations
5. Social Determinants of health-diversity, equity, inclusion priorities

II. Neonatal Neurology

A. Neurodiagnosis
1. Serial neurologic examinations
2. EEG – vEEG, aEEG
3. Cranial ultrasound and Doppler studies
4. CT scan
5. MRI/MRA/MRV
6. MRS
7. NIRS
8. Placental-cord-uterine pathology
9. Neurogenetics

B. Brain development (embryonic-fetal stages)
1. Expected neural development by trimester
2. Neocortical and hindbrain malformations
3. Spinal cord malformations
4. Anomalous versus destructive disease pathways related to exposome effects

C. Neonatal seizures
1. Classification
2. Etiology
3. Evaluation
4. Therapy
5. Early onset epileptic encephalopathies
6. Risk of postnatal epilepsy

D. Neonatal brain injuries
1. Trauma including epidural, subdural and subgaleal hemorrhages; fractures
2. Preterm injury including GMH, IVH, PVL, WMI, PHVD
   a. Prevention of IVH
   b. PHVD – monitoring ventricular size and treatment

3. Term brain injury
    a. Hypoxic ischemic encephalopathy
    b. Intracranial hemorrhage
4. Infections
    a. Congenital pathogen overview
    b. Bacterial
    c. Viral
    d. Protozoan

E. Stroke
1. Incidence
2. Classification
3. Etiology
4. Evaluation
5. Treatment

F. Neurogenetics
1. Chromosomal disorders
2. Neurocutaneous disorders
3. Inborn errors of metabolism
4. Mitochondrial disorders
5. Neuromuscular disorders
6. Neurodegenerative disease
7. Testing tools: microarray, whole exome sequencing, genome-wide association studies
8. Post-translational genetic advances e.g. epigenetic mechanisms, imprinting somatic mosacism

G. Neuromuscular
1. Neonatal hypotonia
2. Congenital myopathies
3. Myotonic dystrophy
4. Metabolic myopathies
5. Arthrogryposis – fetal dyskinesia syndromes
6. Evaluations using neurodiagnostic testing

H. Neuroprotection
1. Neuromonitoring applications
    a. NIRS
    b. EEG
2. Therapeutic hypothermia/ erythropoietin
3. IVH prevention bundle
4. Pharmacologic agents for primary/secondary/tertiary forms of neuronal death

I. Systemic disorders (principal examples)
1. Complex congenital heart disease
2. Pulmonary hypertension

3. Bronchopulmonary dysplasia complicated by chronic lung disease
4. Sepsis/fetal inflammatory response syndrome
5. Necrotizing enterocolitis

J. Other toxic or traumatic disorders
1. Toxic stressor interplay involving endogenous/exogenous sources
2. Pharmacologic exposures including neuropsychiatric medications
3. Neonatal abstinence syndrome
4. Neonatal hyperbilirubinemia

K. Pain management and analgesia/anesthesia exposure

II. Fetal neurology

A. Preconception planning
1. Contributions from childhood doseases/adversities
2.Preimplantation testing for reproductive health or disease
3.Fertility issues with testing
4.Artificial reproductive technologies (e.g., IVF)

B. Levels of maternal care
1. ACOG recommendations
2. Integrated roles of OB team members: nurse practitioner, midwife
3.Contributions of a family Doula

C. Prenatal screening
1. Maternal screening
2. CVS, amniocentesis
3. Fetal ultrasound & doppler studies
4. Fetal MRI

D. Abnormalities of the placenta
1. Anatomy, function
2. Normal trimester-specific development
3. Abnormal pathology (e.g. Amsterdam criteria)

E. Fetal growth relative to maternal-placental-fetal triad health and disease
1. Normative growth curves (e.g., 21st century indices)
2. IUGR, SGA, ponderal index
3. Amniotic fluid dynamics: oligo versus polyhydramnios
4. Brain-placental axis relationships

F. CNS development
1. Neuroembryology topics
2. Transient structures, progenitor cell populations, stage of connectivity
3. Development and functioning of the neurovascular unit.
4. Malformations of the developing neuroaxis
5. Ventriculomegaly and other specific structural markers (e.g. absent septum pellucidum)

G. fetal functional neurodevelopment
1. Fetal swallowing
2. Prechtl movement scores
3. Fetal dyskinesia syndrome

H. Fetal seizures
    1. Early infantile epileptic encephalopathies
    2. Genetic/acquired integrated etiologies.
I. Infections
    1. Pathogen specific communicative types of fetal inflammatory response (FIR)
    2. Non-communicative type of FIR
J. Maternal conditions before and during pregnancy
    1. Childhood diseases and adverse experiences
        a. primary neurological: intellectual disabilities, epilepsies, specific genetic disorders, multiple sclerosis, neuromuscular disorders
        b. systemic disorders: e.g. diabetes, hypertension, cardiac, obesity
    2.Preganancy-related disease pathways (e.g., hypertension, diabetes etc.)
    3. Prescribed medication/substance use affecting fetal neurodevelopment
    4.Diagnostic Statistical Manual (DSM) for relevant neuropsychiatric disorders
K. Early onset neurodegenerative disorders
L. Brain tumors
M. End of life issues
N. Outcomes and disabilities
O. Fetal therapies
    1. Infections
    2. Autoimmune
    3. Fetal surgery: systemic and neurosurgery
    4. Exit strategies: e.g. cystic hygroma

III. Comprehensive Care of the high-risk neonate
A. Hearing
B. Vision
C. Standardized developmental assessments
    1. Domains to test
    2. Testing instruments-age-specific
D. Outcomes & disabilities (childhood to adulthood)
    1. Intellectual disability
    2. Neurobehavioral/psychiatric disorders
    3. Executive function disorder and other specific cognitive deficits
    4. Visual changes including cortical visual impairment (e.g., CVI)
    5. Hearing deficits
    6. Sensory integration disorders
    7. Neurologic disorders associated with reproductive senescence.
E. Therapies and rehabilitation
    1. Neonatal
    2. Post-discharge; age specific
    3. Shared Clinical Decisions
    4. Neuropalliative Care

*Modified from reference [3]
